# Diastolic contributors in cardiomyocytes of a cardiometabolic HFpEF-like mouse model

**DOI:** 10.1085/jgp.202614028

**Published:** 2026-08-25

**Authors:** Arooj Shahid, Timothy S. McMillen, Samuel Daugherty, Anna E. Burns, Marloes van den Berg, Michael Regnier, Henk Granzier, Mei Methawasin

**Affiliations:** 1 Department of Medical Pharmacology and Physiology, University of Missouri, Columbia, MO, USA; 2 Department of Anesthesiology and Pain Medicine, Center of Translational Muscle Research, Institute for Stem Cell and Regenerative Medicine, University of Washington, Seattle, WA, USA; 3 Department of Cellular and Molecular Medicine and Sarver Molecular Cardiovascular Research Program, University of Arizona, Tucson, AZ, USA; 4 Department of Bioengineering, University of Washington, Seattle, WA, USA; 5 Center for Translational Muscle Research, University of Washington, Seattle, WA, USA; 6 Institute For Stem Cell and Regenerative Medicine, University of Washington, Seattle, WA, USA

## Abstract

Three main contributors to cardiomyocyte diastolic stiffness are (1) passive sarcomere stiffness, (2) microtubules, and (3) diastolic crossbridges (XBs), the XBs that are present in the diastolic phase. However, the relative contributions of these key determinants in heart failure with preserved ejection fraction (HFpEF) conditions are unclear. We quantify the relative contributions of passive sarcomere stress, the microtubule network, and diastolic XB activity to overall diastolic stress in intact cardiomyocytes isolated from two-hit mice, a cardiometabolic HFpEF-like model, in both sexes. The stretch-release protocol was used to obtain the diastolic stress–sarcomere length relation. XB inhibitor and colchicine treatment were used to determine the contributions of diastolic XBs and microtubules, respectively. Passive sarcomere stress was measured in cells treated with both colchicine and the XB inhibitor. Male HFpEF-like cardiomyocytes exhibit increases in both passive sarcomere stress (by 70%) and diastolic XBs (by 52%), whereas female HFpEF-like cardiomyocytes show an increase in passive sarcomere stress alone (by 55%). The microtubule network contributes to diastolic stress by augmenting the extent of diastolic XBs in males. The elevated diastolic XBs in male HFpEF-like mice are accompanied by altered Ca^2+^ transient, suggesting that remodeled Ca^2+^ handling accounts, in part, for the enhanced diastolic XB activity in males. These findings imply that, in males, both increased passive sarcomere stiffness and diastolic XB activity represent potential therapeutic targets for reducing cardiomyocyte diastolic stiffness, whereas in females, diastolic stiffness is predominantly driven by elevated passive sarcomere stiffness.

## Teaser

In the cardiomyocytes of male two-hit mice, both increased passive sarcomere stiffness and diastolic crossbridge activity represent potential therapeutic targets for reducing cardiomyocyte diastolic stiffness; whereas in females, cardiomyocyte diastolic stiffness is predominantly driven by elevated passive sarcomere stiffness.

## Introduction

The current paradigm of heart failure with preserved ejection fraction (HFpEF) pathophysiology is the manifestation of a chronic systemic inflammatory state (Dunlay et al., 2017; Schiattarella et al., 2021b; Shah et al., 2016; Schiattarella et al., 2021c). In the heart, the inflammatory cytokines cause deleterious effects on various organelles of the cardiomyocytes, including but not limited to the protein quality control system (Schiattarella et al., 2019; Schiattarella et al., 2021a), mitochondria (Tong et al., 2021; Valero-Munoz et al., 2022; Liu et al., 2022; Deng et al., 2021), Ca^2+^-handling proteins (Hegyi et al., 2026; Mira Hernandez et al., 2025; Sequeira et al., 2025; Frisk et al., 2021), cytoskeleton (Eaton et al., 2024; Caporizzo et al., 2020), and sarcomeres.

Cardiac dysfunction in HFpEF is predominantly characterized by impairment of the diastolic phase of the cardiac cycle (Pfeffer et al., 2019; Redfield, 2016; Zile et al., 2004; Nagueh, 2021; Shah et al., 2016; Borlaug, 2020), manifested as delayed myocardial relaxation and increased left ventricular (LV) diastolic stiffness (van Heerebeek et al., 2012a; Borlaug and Paulus, 2011; Paulus and Tschope, 2013).

LV diastolic stiffness arises from cardiomyocytes and the extracellular matrix (Zile et al., 2015). Cardiomyocytes play a central role in regulating both LV relaxation and chamber distensibility. Three main contributors to cardiomyocyte diastolic stiffness are (1) passive sarcomere stiffness, mainly from titin (Chung and Granzier, 2011; Granzier and Irving, 1995), (2) microtubules (Robison et al., 2016; Chen et al., 2018), and (3) diastolic crossbridges (XBs), the XBs that are present in the diastolic phase (Selby et al., 2011; King et al., 2011).

Titin is a giant myofilament protein that spans the sarcomere, extending from one Z-disc to the opposite Z-disc, with an I-band segment that functions as a molecular spring (Watanabe et al., 2002; Li et al., 2002; Granzier and Irving, 1995). Titin’s mechanical stiffness can be modulated by altering titin isoform expression and posttranslational modifications (PTMs) (Chung et al., 2013; Hidalgo and Granzier, 2013; LeWinter and Granzier, 2010). Importantly, titin’s stiffness is increased in human HFpEF myocardium (Zile et al., 2015; Hopf et al., 2018).

Microtubules are cytoskeletal structures that are not components of the sarcomere itself but anchor to the sarcomere and function as a load-bearing element, providing mechanical resistance during sarcomere shortening and lengthening (Robison et al., 2016; Kerr et al., 2015; Robison and Prosser, 2017; Kreitzer et al., 1999). Microtubules are upregulated and undergo detyrosination, a PTM that increases their stiffness, in the myocardium of human end-stage heart and a rat model of HFpEF (Chen et al., 2018; Tsutsui et al., 1993; Eaton et al., 2024; Caporizzo et al., 2020). Restoration of tubulin tyrosination improves diastolic function in in vivo animal models and isolated human cardiomyocytes (Chen et al., 2020; Pietsch et al., 2024; Eaton et al., 2024).

Diastolic XBs are the XBs that remain throughout diastole, which could lead to incomplete LV relaxation and limited LV distensibility in pathological conditions (Donaldson et al., 2012). Elevated diastolic XB activity during tachycardia contributes to reduced LV filling volume and exercise intolerance in patients with hypertrophic cardiomyopathy (HCM) (Selby et al., 2011; King et al., 2011; Runte et al., 2017; Donaldson et al., 2012; Sequeira et al., 2015a).

In addition to these primary contributors, intermediate filaments such as desmin, which anchor the microtubule network to the Z-disc region of the sarcomere, and the sarcolemma also contribute to overall diastolic mechanical properties (Loescher et al., 2023). However, the relative contributions of these contributors in HFpEF conditions remain unclear.

In the present study, we used the two-hit mouse model, a cardiometabolic HFpEF-like model (Schiattarella et al., 2019), to determine which component contributes most significantly to diastolic stress and may therefore represent a potential therapeutic target for modifying cardiomyocyte diastolic stiffness in HFpEF conditions. Adult wild-type female mice are relatively resistant to HFpEF development (Tong et al., 2019; Methawasin et al., 2025b), and most women with HFpEF are in a postmenopausal state (Shah et al., 2020; Sabbatini and Kararigas, 2020; Mishra and Kass, 2021). Therefore, in female mice, we first induced an ovary-intact postmenopausal state and subsequently applied the two-hit protocol to induce HFpEF-like conditions. Our previously published work (Methawasin et al., 2025b) demonstrated that the ovary-intact postmenopausal two-hit female mice developed HFpEF-like phenotypes, characterized by diastolic dysfunction at both the LV and the single-cell levels, along with a reduction in transcription levels of spliced X-box–binding protein 1 (Xbp1s), an important unfolded protein response that has been observed to be suppressed in human HFpEF myocardium (Schiattarella et al., 2019).

We first measured total diastolic stress in isolated intact cardiomyocytes. We then physiologically dissected the relative contributions of diastolic XBs, the microtubular network, and passive sarcomere stiffness by pharmacologic intervention. Colchicine was administered to depolymerize microtubules, and butane-2,3-dione monoxime (BDM) was used to inhibit XB interactions. We refer to diastolic stress after XB inhibition as non-XB stress, and diastolic stress after combined colchicine and XB inhibition as passive sarcomere stress. The microtubular contribution was calculated as the difference in diastolic stress between colchicine-treated and untreated cells. The XB contribution was determined from the reduction in diastolic stress following XB inhibition. Passive sarcomere stress was measured in cells treated with both colchicine and the XB inhibitor.

We observed sex differences in the contributors to diastolic stress. Male HFpEF-like cardiomyocytes exhibit increases in both passive sarcomere stress and diastolic XBs, whereas female HFpEF-like cardiomyocytes shows an increase in passive sarcomere stress alone. The microtubule network contributes to diastolic stress by augmenting the extent of diastolic XBs in males. The elevated diastolic XBs in male HFpEF-like are associated with altered Ca^2+^ transients. Collectively, these findings imply that, in males, both increased sarcomere passive stiffness and diastolic XB activity represent therapeutic targets, whereas, in females, diastolic stiffness is predominantly driven by elevated passive sarcomere stiffness.

## Materials and methods

### Experimental animals

Adult male and female C57BL/6N mice were used. All procedures were performed according to the NIH Guide for the Care and Use of Laboratory Animals and approved by the Institutional Animal Care and University Committee of the University of Arizona.

Ovary-intact menopause was induced in a subgroup of female mice by 4-vinylcyclohexene dioxide (VCD) intraperitoneal injection at 160 mg/kg daily for 20 days (Konhilas et al., 2020; Brooks et al., 2016). VCD injection began at 6 wk of age. Vaginal cytology was conducted daily to determine the onset of menopause, defined as 10 consecutive days of diestrus (Konhilas et al., 2020; Brooks et al., 2016). The other subgroup of females received a sesame oil injection, the solvent of VCD, and remained in pre-menopausal state. After the VCD-treated female mice entered menopause (menopause occurred between days 50 and 60 after the initiation of VCD injection [Konhilas et al., 2020; Brooks et al., 2016]), they were subjected to a two-hit regimen to induce metabolic HFpEF (Schiattarella et al., 2019). Male mice were subjected to a two-hit regimen, without any modifications to their sex hormones.

### HFpEF-like condition was induced using a two-hit regimen (Schiattarella et al., 2019)

The two-hit protocol was initiated in both male and female mice at 3.5 mo of age. For the two hit, mice were fed with a high-fat diet (D12492; Research Diets Inc.) and 0.5g/L Nω-Nitro-L-arginine methyl ester (L-NAME; nitric oxide synthase inhibitor) in drinking water, continuing for 4 mo. Control mice were fed a control diet (D12450K, Research Diets Inc.) and regular water. Mice were divided into four groups: (1) male-ctrl diet, (2) male-two-hit, (3) female-pre-menopause-ctrl diet, and (4) female-postmenopause (VCD)-two-hit. For simplicity, we refer to these groups as male-ctrl, male HFpEF-like, female-ctrl, and female HFpEF-like, respectively.

### Loaded intact cardiomyocytes

Cells were isolated as described previously (O’Connell et al., 2007). Briefly, mice were heparinized (1,000 U/kg, i.p.) and euthanized using isoflurane. The heart was removed and cannulated via the aorta with a blunted 21-gauge needle for antegrade coronary perfusion. The heart was perfused with perfusion buffer ([in mmol/L] 90 NaCl, 34.7 KCl, 0.6 KH_2_PO_4_, 0.6 Na_2_HPO_4_, 1.2 MgSO_4_, 12 NaHCO_3_, 10 KHCO_3_, 10 HEPES, 10 taurine, 5.5 glucose, 5 BDM, and 20 creatine, pH 7.4), followed by 0.05 mg/ml Liberase TM (Roche Applied Science). All intact cell experiments were performed at 37°C in Medium 199 plus 10 μg/ml insulin. An inverted microscope (IX-70; Olympus) was used with a chamber with platinum electrodes to electrically stimulate cells and a perfusion line with heater control and suction out to maintain a ∼2 ml/min flow rate. Cells were field stimulated at 4 Hz by MyoPacer stimulator (IonOptix LLC). All images were recorded with a 40× objective lens. Data were collected using an IonOptix FSI A/D board and IonWizard 6.4 software (IonOptix LLC). The glass rods coated with myotak (IonOptix LLC) were carefully lowered onto opposite ends of the cell. The myocyte was attached at one end to a glass rod that connected to the force transducer (OFT200, OptiForce transducer, IonOptix LLC). The other end of the cell was attached to a glass rod connected to the piezo translator (Mad City Lab). The distance between the two glass rods was maintained at ∼80 µm for all cells. The cross-sectional area of the intact cell was obtained from the measured cell width, assuming that the cell’s cross section was an ellipse (Granzier and Irving, 1995). All forces were normalized to stress. Data analysis was performed in IonWizard software 7.0.

### BDM (Backx et al., 1994)

To inhibit actomyosin XB interactions, BDM was dissolved in Medium 199 at a final concentration of 20 mM and perfused into the cell chamber during measurement.

### Four-stretch protocol

**Figure S1.**
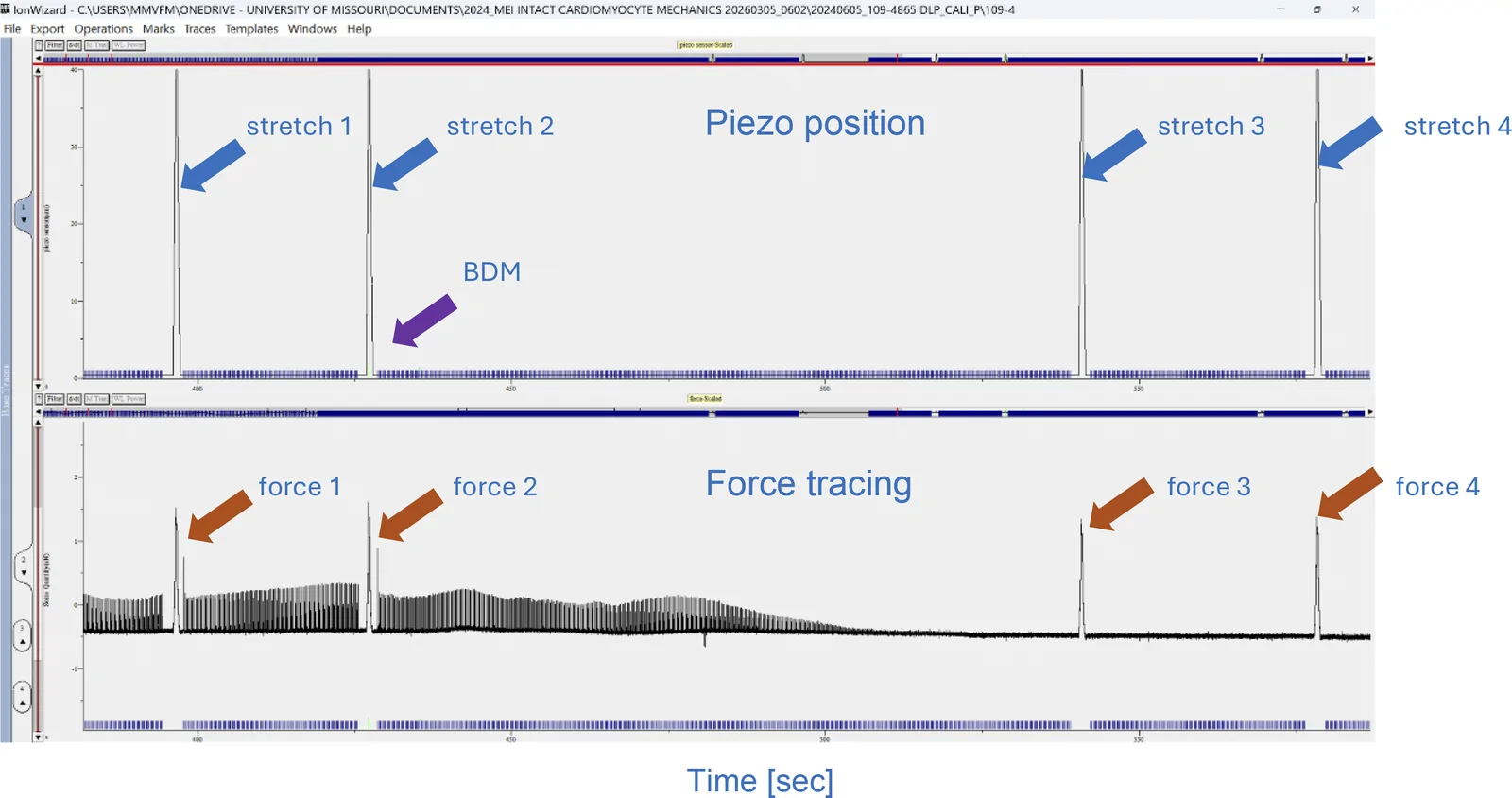
Representative tracings from the four-stretch protocol.

To assess the extent of diastolic XBs, a total of four trapezoid stretches were applied using the same stretch amplitude, as illustrated in Fig. S1. The first stretch was used to ensure that the sarcomere length (SL) reached ≥2.15 μm and to verify secure cell attachment. The second stretch was used to measure force before BDM treatment. BDM was then introduced into the perfusion solution, and the third stretch was performed to measure force after BDM treatment. Finally, the fourth stretch was performed to confirm that cell attachment remained stable throughout the experiment. The stress reduction after BDM treatment represented the extent of diastolic XB stress. A reduction in stress during the fourth stretch compared with the third stretch was considered indicative of loosening of the attachment, and those cells were excluded from analysis.

### Colchicine treatment

To depolymerize microtubules, cells were divided into two groups: one group was incubated with 10 µM colchicine for 90 min, while the non-colchicine group was incubated with DMSO, the solvent for colchicine. Following incubation, cells were used for force measurements, and 10 µM colchicine diluted in Medium 199 was continuously perfused into the experimental chamber during recording.

### Measurement of Ca^2+^ in unloaded intact cardiomyocytes

Isolated LV cardiomyocytes were incubated with 2 µM of Fura-2 AM (F-1225; Life Technologies) for 10 min at room temperature and resuspended in Medium 199 (M5017; Sigma-Aldrich). Fura-2 was excited alternately at 340 and 380 nm, and emission was recorded at 510 nm. Background fluorescence was subtracted for each excitation wavelength. The ratio of fluorescence intensities excited at 340 and 380 nm was used as a relative measurement of cytoplasmic Ca^2+^.

### Myofibril mechanics

Myofibril activation and relaxation measurements were performed on a custom setup as previously described (Racca et al., 2016). Frozen mouse LV tissue was demembranated overnight at 4°C in relaxing solution containing (in mM) 100 KCl, 10 MOPS, 5 EGTA, 9 MgCl_2_, 4 NaATP, 50% glycerol (by volume), and 1% Triton X-100. The demembranated muscle tissue was then transferred to a 50% glycerol-relaxing solution and stored at −20°C for use up to 2 days. To obtain isolated myofibrils, small pieces of muscle tissue were placed into rigor solution (in mM): 50 Tris, 100 KCl, 2 MgCl_2_, 1 EGTA, and 2 dithiothreitol, pH 7, containing a 1:200 dilution of protease inhibitor cocktail (Sigma-Aldrich, P8340). After two rinses in rigor solution, the tissue was shredded with a high-speed homogenizer for one or two bursts of 10 s. Myofibrils were stored at 4°C and used within 2 days. Myofibrils were then plated onto the custom setup and were mounted between a glass force transducer and an inflexible motor arm. A dual photodiode system measured myofibril force by recording needle deflection. The force transducer needle stiffness measured 8.7 μm/μN. A double-barreled glass pipette delivered relaxing (pCa = 9.0), maximal and submaximal activating (pCa = 4.0, 5.6, respectively) solutions to the isolated myofibril using a rapid switching technique. Activation and relaxation data were collected at 15°C and fitted with either single-exponential curves or linear coefficients.

### Statistics

Statistical analysis was performed in SPSS and GraphPad Prism 10. The data are presented as means ± SE, with P values indicated above the lines connecting the compared groups. Statistical significance was set at P < 0.05, *P ≤ 0.05, **P ≤ 0.01, ***P ≤ 0.001, and ****P ≤ 0.0001. The estimated sample sizes were calculated by power analysis using G*Power version 3.1.9.7. The normality of data was tested with the D’Agostino and Pearson and Shapiro–Wilk tests. Homogeneity of variance was tested with Brown–Forsythe and Bartlett’s test or F-test. Outliers were identified using the ROUT method with a Q-value of 10%. A linear mixed model (SPSS) was used for analysis to account for cells or myofibrils clustered within individual animals. The main effects (sex and heart condition) and the interaction of the main effects (sex*heart condition) were analyzed, followed by the least significant difference comparison test. Simple linear regression was used to analyze the slopes of XB–SL relations (Fig. 3, A and B). Pearson correlation analysis was used to examine the relationship between XB stress and passive sarcomere stress (Fig. 6 C).

### Online supplemental material

Fig. S1 shows representative tracings from the four-stretch protocol. Table S1 shows the echocardiographic parameters.

## Results

### Diastolic XB stress is elevated in cardiomyocytes from male HFpEF-like mice, but not in those from female HFpEF-like mice

**Figure 1.**
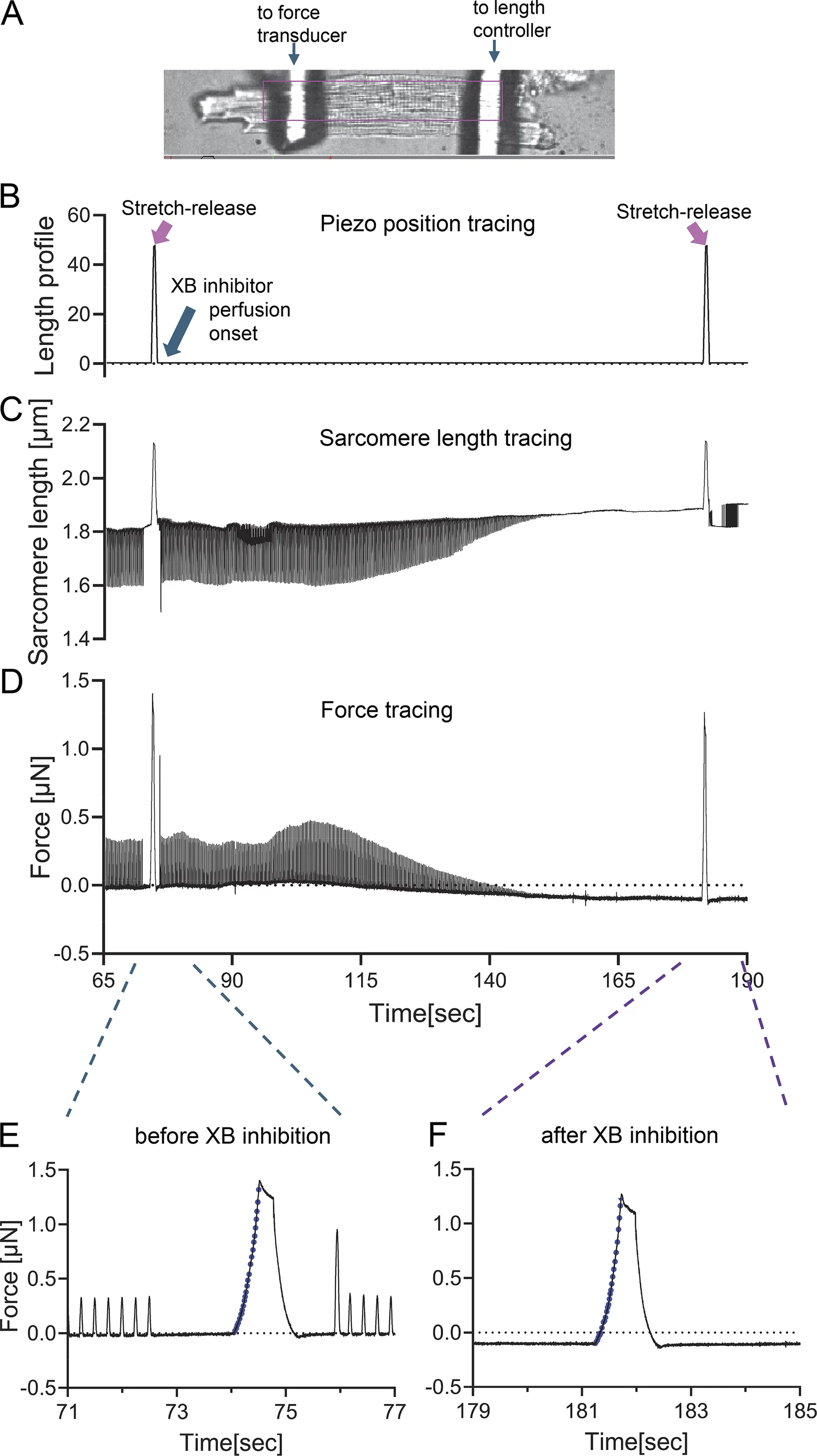
**Experimental approach to measure diastolic XBs in intact cardiomyocytes.** All intact cell experiments were performed at 37°C, and cells were stimulated at 4 Hz. **(A)** An intact cardiomyocyte was attached to the glass rods that connected to the piezo length controller and the force transducer. **(B)** A trapezoid stretch-release protocol was applied during a prolonged diastolic interval (pacing paused) to obtain the force–SL relation. The cell was then perfused with 20 mM BDM, a XB inhibitor. The stretch-release was repeated after contraction had completely stopped. **(C and D)** Representative tracings of SL (C) and force (D) show that, following XB inhibition, the cell gradually ceased beating, accompanied by a progressive increase in baseline SL and a decrease in baseline force. **(E and F)** Show zoomed-in force tracings before and after XB inhibition. The difference in up-ramp force (highlighted in blue) measured before and after XB inhibition was used to calculate the extent of diastolic XBs.

To quantify the contribution of diastolic XBs to total diastolic stress, we measured diastolic stress in intact cardiomyocytes before and after administration of BDM, an XB inhibitor. As shown in Fig. 1 A, an isolated cardiomyocyte was attached with adhesive to glass rods connected to a force transducer and a length controller. Experiments were conducted in a chamber continuously perfused with Medium 199, maintained at 37°C, and electrically paced at 4 Hz.

A trapezoidal stretch-release protocol was applied during a prolonged diastolic interval (with pacing temporarily paused; Fig. 1 B). Cells were stretched at 1 µm/msec to a target SL of ≥2.15 µm. This target SL lies within the physiological diastolic SL range of the mouse left ventricle (∼1.8–2.2 µm) (Chung and Granzier, 2011; Kobirumaki-Shimozawa et al., 2018). The tracings of the length controller position (Fig. 1 B), the SL (Fig. 1 C), and the force (Fig. 1 D) were recorded over time.

To assess the extent of diastolic XBs, the perfusion solution was then switched to Medium 199 containing 20 mM BDM, an XB inhibitor (Fig. 1 B), which inhibits the rate of phosphate release, stabilizing the myosin-ADP-Pi state of the ATPase cycle (Backx et al., 1994; Herrmann et al., 1992). After BDM was perfused, the cells gradually lost contractile force and eventually stopped beating. Complete cessation of contraction occurred after ∼100–120 s. Once contractions had fully ceased, the stretch-release protocol was repeated (Fig. 1 B).

Representative long recordings demonstrate a progressive increase in diastolic SL (Fig. 1 C), accompanied by a decrease in baseline force (Fig. 1 D). This occurs because the XBs detach, allowing titin’s restoring force to push the Z-disc away from the sarcomere center, and setting a new baseline SL (Helmes et al., 1996; King et al., 2011). The force corresponding to the up-ramp phase of the trapezoid stretch was used to determine the diastolic stress–SL relation (blue lines in Fig. 1, E and F). Diastolic force measured in the presence of the XB inhibitor (Fig. 1 F) is reduced compared with the force measured prior to inhibition (Fig. 1 E). All forces were normalized by cellular cross-sectional area to stress.

**Figure 2.**
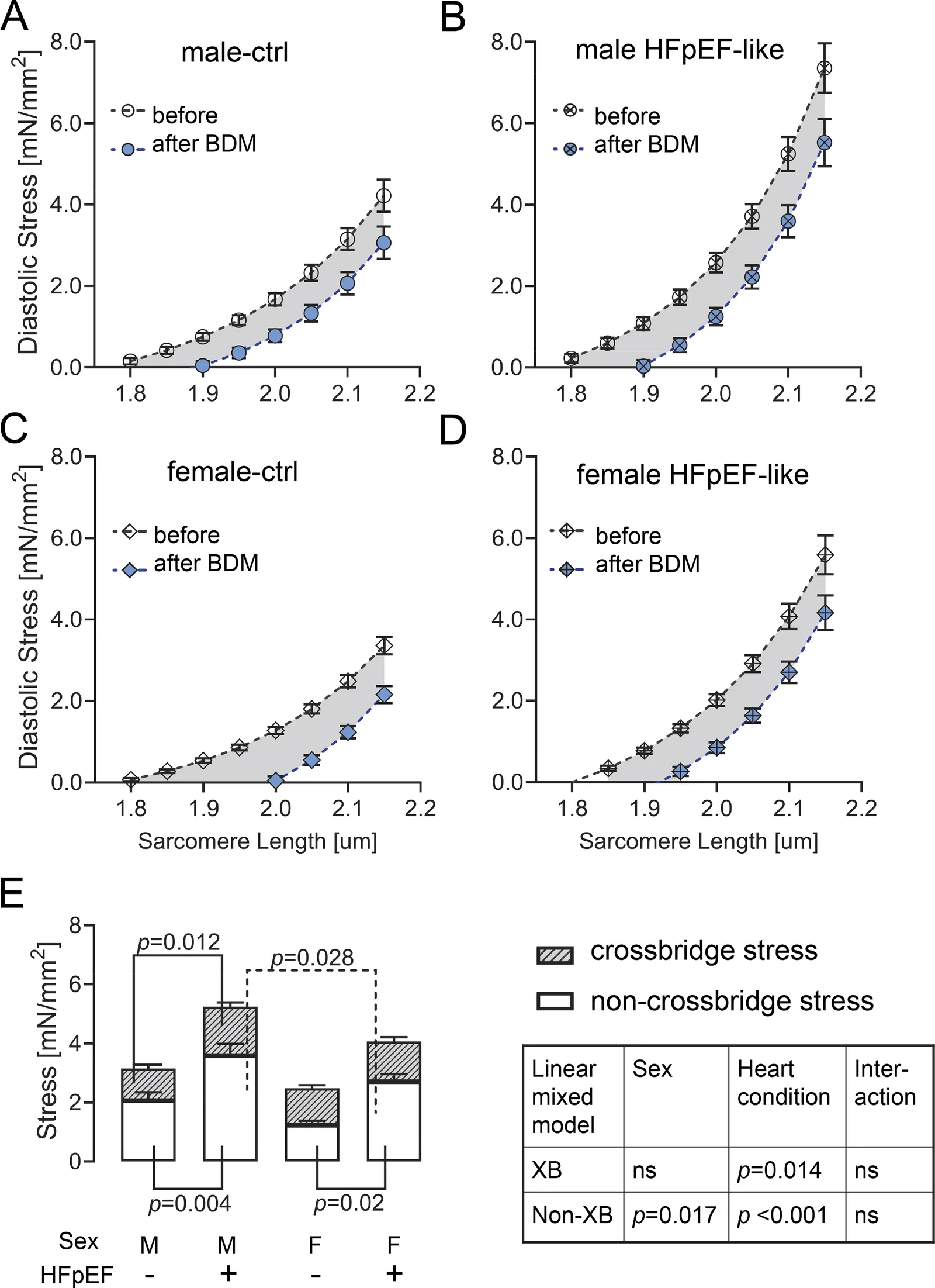
**Diastolic XBs contribute to diastolic stress in intact cardiomyocytes. (A–D)** Show diastolic stress over SLs in male-ctrl, male HFpEF-like, female-ctrl, and female HFpEF-like, respectively. The difference in diastolic stress measured before and after treatment with the XB inhibitor BDM (gray area) represents the extent of XB contribution. **(E)** The contributions of XB and non-XB stress at SL of 2.15 µm are summarized in E, and the significance of main effects (sex and heart condition) is shown. The data are presented as means ± SE. The sample sizes are 11, 13, 10, and 12 mice for male-ctrl, male HFpEF-like, female-ctrl, and female HFpEF-like, with 2–4 cells analyzed per mouse. Analysis was performed using a linear mixed model with least significant difference post-hoc comparisons. ns indicates not significant. For simplicity, post-hoc comparisons between male-ctrl vs. female HFpEF-like and male HFpEF-like vs. female-ctrl are not shown.

Fig. 2 shows the diastolic stress–SL relations before and after XB inhibition in male-ctrl (Fig. 2 A), male HFpEF-like (Fig. 2 B), female-ctrl (Fig. 2 C), and female HFpEF-like (Fig. 2 D). Following XB inhibition, baseline SLs are increased (X-intercept is shifted to the right), and diastolic stress is shifted downward relative to pre-inhibition levels. The stress reduction after XB inhibition (Fig. 2, A–D, gray area) represents the extent of diastolic XB stress.

The contributions of XB and non-XB stress at an SL of 2.15 µm are summarized in Fig. 2 E. There is a significant effect of heart condition (ctrl vs. HFpEF-like) on XB stress and significant effects of both sex and heart condition on non-XB stress (Fig. 2 E, right). For non-XB stress, values are higher in male HFpEF-like compared with male-ctrl (∼70%), in female HFpEF-like compared with female-ctrl (∼55%), and in male HFpEF-like compared with female HFpEF-like (∼33%). For XB stress, male HFpEF-like is significantly higher than male-ctrl, whereas no significant difference is observed between female HFpEF-like and female-ctrl.

**Figure 3.**
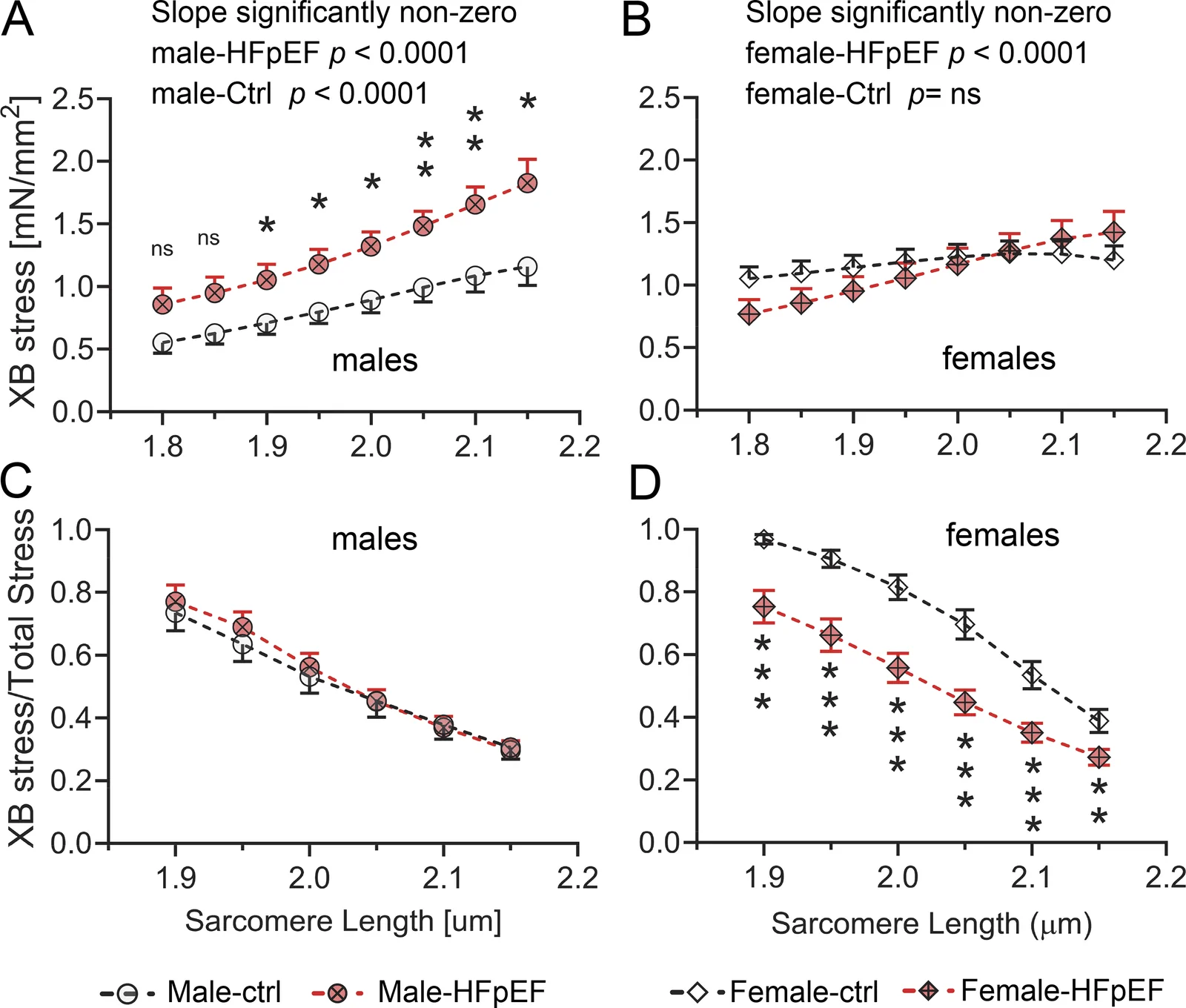
**The extent of diastolic XBs is increased in male HFpEF-like but not in female HFpEF-like cardiomyocytes. (A and B)** XB stress across diastolic SLs in cardiomyocytes from control vs. HFpEF-like males (A) and females (B). **(C and D)** The ratio of diastolic XBs to total diastolic stress across diastolic SLs of control vs. HFpEF-like cardiomyocytes of male (C) and female (D) mice. The data are presented as means ± SE. The sample sizes are 11, 13, 10, and 12 mice for male-ctrl, male HFpEF-like, female-ctrl, and female HFpEF-like, with 2–4 cells analyzed per mouse. Analysis was performed using a linear mixed model at individual SLs. Simple linear regression was used to compare the slopes of the XB–SL relations (A and B). *P ≤ 0.05, **P ≤ 0.01, and ***P ≤ 0.001.

The extent of XB stress across diastolic SLs in males and females is shown in Fig. 3, A and B, respectively. XB stress is elevated in male HFpEF-like compared with male-ctrl across SLs of 1.9–2.15 µm, whereas female HFpEF-like and female-ctrl show no difference. Linear regression analysis shows that the slope of XB stress-SL relation is significantly different from zero in male-ctrl, male HFpEF-like, and female HFpEF-like, whereas the slope in female-ctrl is not different from zero. This indicates that XB stress increases with SL in the first three groups, while it remains relatively constant across the SL range in female-ctrl. The ratio of XB-to-total diastolic stress across SLs (Fig. 3, C and D) suggests that XB stress contributes more at shorter SLs, and this contribution gradually decreases as SL increases, reflecting the rising contribution of non-XB stress. At longer SLs, diastolic stress is primarily driven by non-XB mechanisms. Given that the physiological SL range in the mouse LV is ∼1.8–2.2 μm (Chung and Granzier, 2011), diastolic XBs play a significant role within the operating SL range.

Although absolute XB stress is higher in male HFpEF-like (Fig. 3 A), the XB-to-total stress ratio does not differ between male groups (Fig. 3 C), reflecting increases in both XB and non-XB stress in male HFpEF-like (Fig. 2 E). In contrast, absolute XB stress is similar between female-ctrl and female HFpEF-like (Fig. 3 B), but the XB-to-total stress ratio is higher in female-ctrl across the SLs (Fig. 3 D) due to the increased non-XB stress without an increase in XB stress in female HFpEF-like (Fig. 2 E).

These results indicate that increased diastolic XB interactions in HFpEF occur in males, but not females, and that diastolic XB contributions are particularly important at shorter SLs within the physiological operating range.

### Microtubular network influences the magnitude of diastolic XB in male HFpEF-like mice

To assess the contribution of the microtubular network, a subset of cardiomyocytes was treated with 10 μM colchicine for 90 min to depolymerize microtubules, while another subset was treated with DMSO, the vehicle control (Granzier and Irving, 1995; Stones et al., 2013). Cells were then subjected to diastolic stress measurement using the stretch-release protocol.

**Figure 4.**
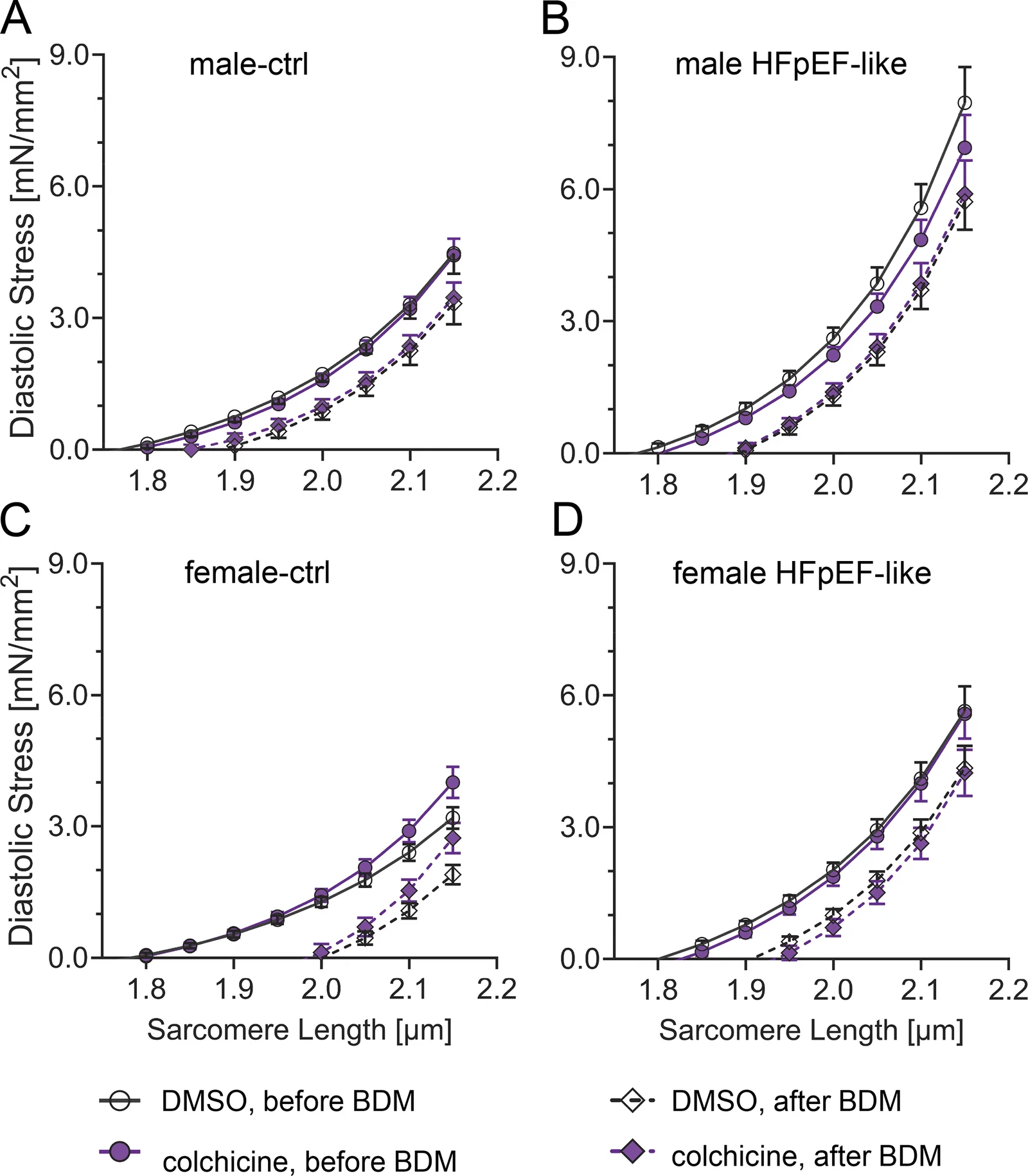
**Contribution of microtubules in intact cardiomyocytes with and without XB inhibition. (A–D)** To evaluate the contribution of the microtubular network, diastolic stress–SL relation was measured in colchicine-treated and DMSO-treated cardiomyocytes, both before and after treatment with BDM, a XB inhibitor in (A) male-ctrl, (B) male HFpEF-like, (C) female-ctrl, and (D) female HFpEF-like mice. No statistically significant effect of colchicine on diastolic stress is observed in any group, although the male HFpEF-like group shows a trend toward reduced diastolic stress before BDM treatment in colchicine compared with DMSO-treated cells. The data are presented as means ± SE. The sample sizes are 9, 10, 8, and 11 mice for male-ctrl, male HFpEF-like, female-ctrl, and female HFpEF-like, with 2–4 colchicine-treated cells and 2–4 DMSO-treated cells analyzed per mouse. Analysis was performed using a linear mixed model at individual SLs. The statistical significance of stress before and after BDM is not shown for simplicity.

The diastolic stress–SL relation was measured before and after treatment with BDM, the XB inhibitor (Fig. 4, A–D, solid lines represent stress measured before BDM, whereas dashed lines represent stress after BDM). No statistically significant effect of colchicine on diastolic stress is observed in any group, although the male HFpEF-like group shows a trend toward reduced diastolic stress in colchicine compared with DMSO-treated cells before BDM treatment.

**Figure 5.**
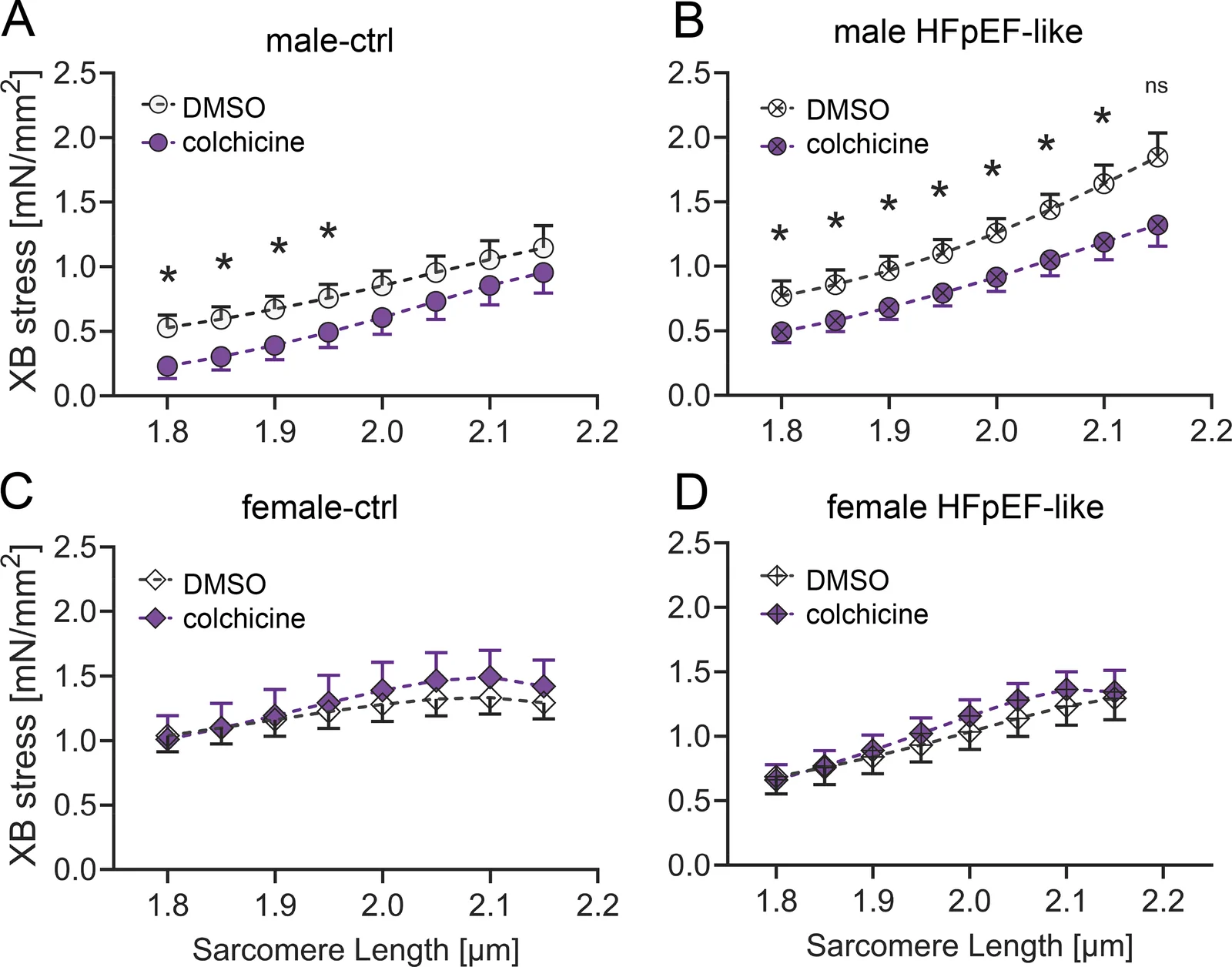
**Microtubules influence the magnitude of diastolic XBs. (A–D)** XB stress across SL is shown for colchicine-treated vs. DMSO-treated cells in four experimental groups: male-ctrl (A), male HFpEF-like (B), female-ctrl (C), and female HFpEF-like (D). Colchicine-treated cells exhibit lower diastolic XB levels than DMSO-treated cells in male HFpEF-like (B) and male-ctrl at shorter SLs (A). The data are presented as means ± SE. The sample sizes are 9, 10, 8, and 11 mice for male-ctrl, male HFpEF-like, female-ctrl, and female HFpEF-like, with 2–4 colchicine-treated cells and 2–4 DMSO-treated cells analyzed per mouse. Analysis was performed using a linear mixed model at individual SLs. *P ≤ 0.05 and ns indicates not significant.

Next, we plotted the extent of diastolic XBs across the SLs of colchicine-treated vs. DMSO-treated cells (Fig. 5, A–D). Colchicine treatment reduces the magnitude of diastolic XB stress in male HFpEF-like over the SL range of 1.8–2.1 μm (Fig. 5 B) and in male-ctrl at shorter SLs (1.8–1.95 μm; Fig. 5 A), with no effect observed in females (Fig. 5, C and D). These findings suggest that microtubules contribute to diastolic stress by enhancing diastolic XB interactions in male mice.

### Passive sarcomere stress is elevated in HFpEF-like cardiomyocytes in both male and female mice

**Figure 6.**
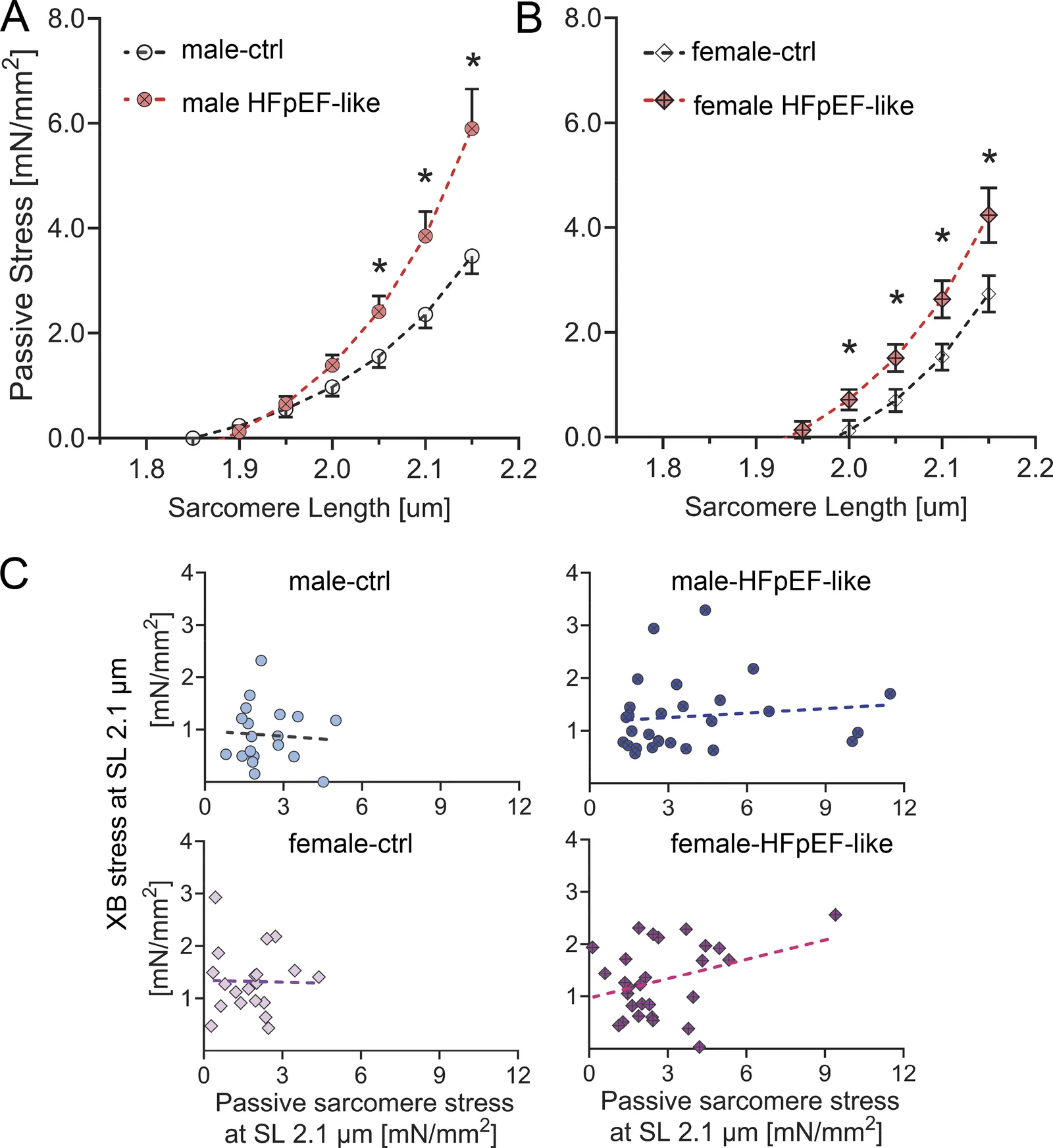
**Passive sarcomere stress is increased in cardiomyocytes from both male HFpEF-like and female HFpEF-like mice. (A and B)** Diastolic stress measured in cardiomyocytes following combined treatment with BDM and colchicine, which inhibit XB activity and depolymerize microtubules, reflects passive sarcomere stress. **(A and B)** Passive sarcomere stress is elevated in cardiomyocytes from male HFpEF-like (A) and female HFpEF-like mice (B) compared with their respective controls. **(C)** The correlation between diastolic XB stress and passive sarcomere stress at a SL of 2.1 µm is plotted for four experimental groups: male-ctrl, male HFpEF-like, female-ctrl, and female HFpEF-like (C). There is no correlation between sarcomere passive stress and the extent of diastolic XB stress. For A and B, the data are presented as means ± SE. The sample sizes are 9, 10, 8, and 11 mice for male-ctrl, male HFpEF-like, female-ctrl, and female HFpEF-like, with 2–4 cells analyzed per mouse. Analysis was performed using a linear mixed model at individual SLs, *P ≤ 0.05. For C, the sample sizes include *n* = 19, 32, 23, and 29 cells from 9, 10, 8, and 11 mice for male-ctrl, male HFpEF-like, female-ctrl, and female HFpEF-like, respectively. Analysis was performed using Pearson correlation. Each data point represents an individual cell.

Passive sarcomere stress was assessed in cells following combined treatment with BDM and colchicine, which inhibit XB activity and depolymerize microtubules, respectively. Passive sarcomere stress is elevated in male HFpEF-like cardiomyocytes compared with male-ctrl starting at a SL of 2.05 µm (Fig. 6 A). In females, passive sarcomere stress in HFpEF-like cardiomyocytes is higher than in female-ctrl starting at SL 2.0 µm, with a shorter baseline SL observed in female HFpEF-like (∼1.94 µm) compared with female-ctrl (∼1.99 µm) (Fig. 6 B).

The increased diastolic stress in male HFpEF-like cardiomyocytes reflects contributions from both passive sarcomere stress (Fig. 6 A) and diastolic XBs (Fig. 3 A), whereas in female HFpEF-like cardiomyocytes, the increase is primarily due to elevated passive sarcomere stress (Fig. 3 B and Fig. 6 B).

### Passive sarcomere stress does not influence the magnitude of diastolic XB stress

Because passive sarcomere stress is primarily determined by titin, and titin stress can regulate length-dependent activation of the myofilament, increased titin stress sensitizes XBs to Ca^2+^, promoting greater XB recruitment and force (Lee et al., 2013; Ait-Mou et al., 2016). Thus, we sought to determine whether passive sarcomere stress influences the diastolic XB level.

XB stress of each cardiomyocyte at SL 2.1 µm was plotted against passive sarcomere stress at the same SL, and correlation analyses were performed for each group (Fig. 6 C). No significant correlation is observed between diastolic XB stress and passive sarcomere stress, including in male HFpEF-like, where both XB stress and passive sarcomere stress are elevated. These findings suggest that passive sarcomere stress does not influence the magnitude of diastolic XB stress.

### There is an alteration in Ca^2+^ handling in male HFpEF-like cardiomyocytes

**Figure 7.**
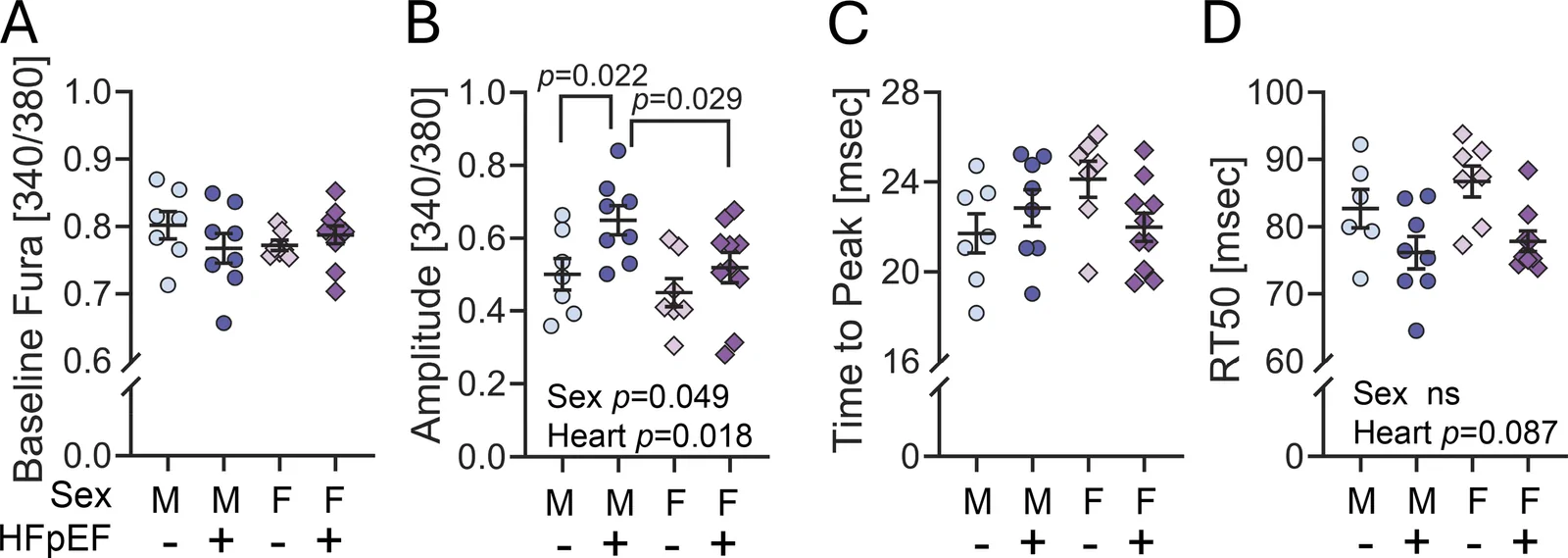
**Ca** ^**2+**^ **handling is altered in cardiomyocytes from male HFpEF-like mice. (A–D)** Ca^2+^ transients in isolated intact cardiomyocytes were measured using Fura-2 under 4 Hz stimulation. The Fura ratio (340/380) at diastole (A), transient amplitude (B), time to peak (C), and time to 50% transient decay (RT50; D) are shown. The data are presented as means ± SE. The sample sizes include *n* = 334, 337, 226, and 483 cells from 7, 8, 7, and 10 mice for male-ctrl, male HFpEF-like, female-ctrl, and female HFpEF-like. Analysis was performed using a linear mixed model with least significant difference post hoc comparisons. Main effects are shown only when they are statistically significant. Each data point represents the average value of one mouse. For simplicity, post hoc comparisons between male-ctrl vs. female HFpEF-like and male HFpEF-like vs. female-ctrl are not shown.

Because defects in Ca^2+^ handling can also influence XB interactions, we performed Ca^2+^ release-reuptake studies using Fura-2 in unloaded, intact cardiomyocytes. Baseline fluorescence ratio (340/380), reflecting diastolic Ca^2+^ levels (Fig. 7 A); fluorescence transient amplitude, indicating the amount of Ca^2+^ released in systole (Fig. 7 B); time to peak fluorescence, representing the rate of Ca^2+^ release (Fig. 7 B); and time to 50% fluorescence decay (RT50), reflecting the rate of Ca^2+^ reuptake (Fig. 7 D), are shown. Both sex and heart condition significantly affect transient amplitude (Fig. 7 B), with males exhibiting higher amplitudes than females and the HFpEF-like condition showing higher amplitudes than ctrl. Male HFpEF-like cardiomyocytes show higher amplitudes than male-ctrl, while no differences are observed between two groups of females (Fig. 7 B). Heart condition also significantly affects RT50, with a trend toward rapid Ca^2+^ reuptake in male and female HFpEF-like, although post hoc comparisons are not significant (Fig. 7 D). No significant differences are observed among groups for diastolic Ca^2+^ levels (Fig. 7 A) or Ca^2+^ release kinetics (Fig. 7 C).

### There is no alteration in XB turnover rate or relaxation kinetics at the myofibril levels in HFpEF-like mice

**Figure 8.**
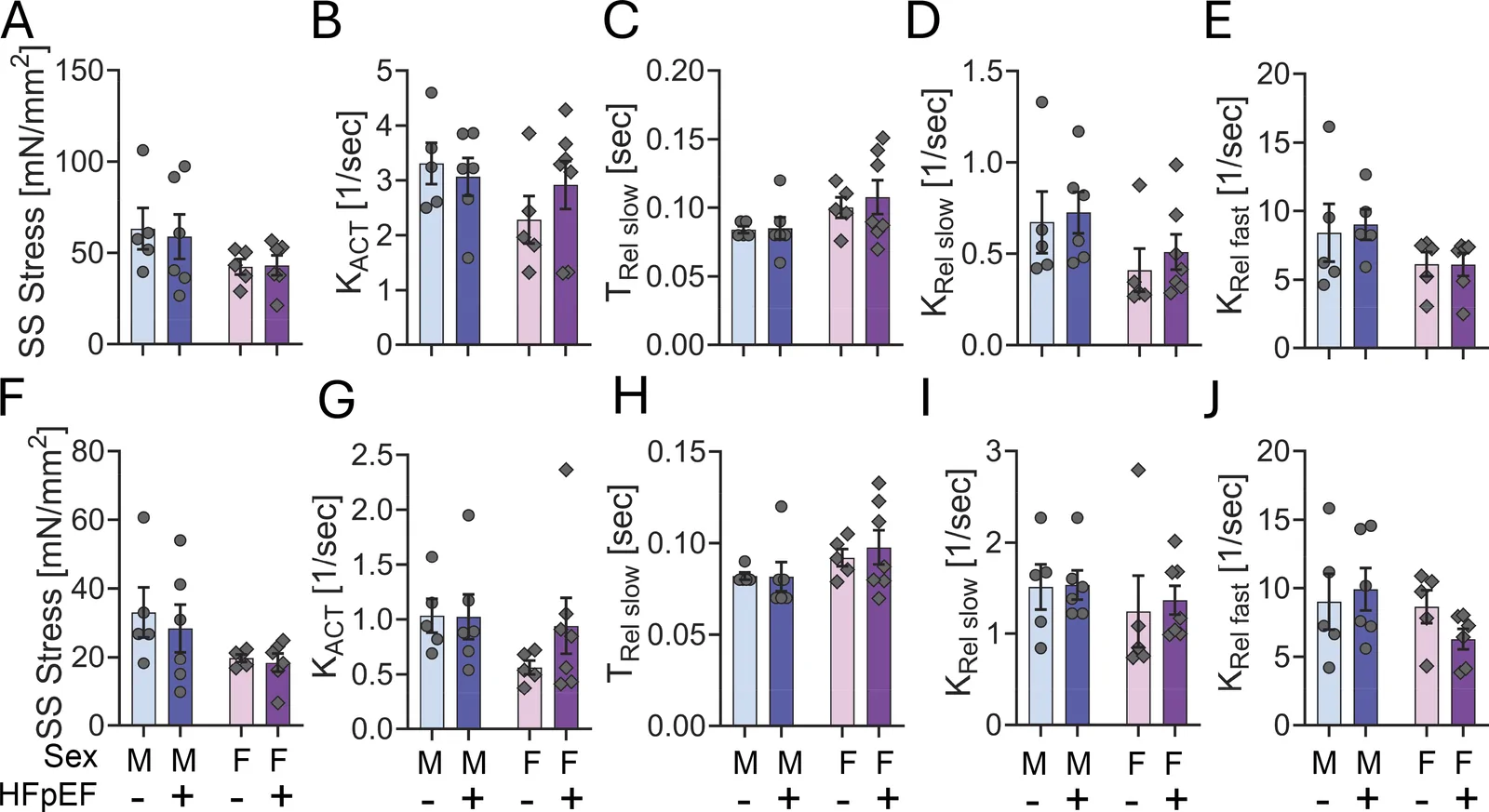
**XB kinetics at the myofibril level are unchanged in HFpEF-like mice. (A–J)** The upper panel (A–E) show cardiac myofibril mechanic parameters at pCa 4, while the lower panels (F–J) show the same parameters at pCa 5.6. Displayed are steady-state stress (A and F), rate constant of force development (B and G), duration of the slow phase of relaxation (C and H), rate constant of the slow phase of relaxation (D and I), and rate constant of the fast phase of relaxation (E and J). The data are presented as means ± SE. The sample sizes include *n* = 5, 6, 5, and 7 mice, with 3–7 myofibrils analyzed per mouse. Analysis was performed using a linear mixed model with least significant difference post hoc comparisons. Each data point represents the average value of one mouse. k_ACT_, rate constant of force development; T_REL,slow_, duration of the slow phase of relaxation; k_REL,slow_, rate constant of the slow phase of relaxation; k_REL,fast_, rate constant of the fast phase of relaxation.

To determine whether changes in XB kinetics underlie the increased diastolic XB stress observed in male HFpEF-like mice, we performed cardiac myofibril mechanics studies. Fig. 8 upper panels (A–E) present XB kinetic studies at maximal Ca^2+^ activation (pCa 4), while Fig. 8 lower panels (F–J) show XB kinetic studies at submaximal Ca^2+^ activation (pCa 5.6).The steady-state force (ssF) at pCa 4 (Fig. 8 A) reflects maximal force-generating capacity; the ssF at pCa 5.6 (ssF; Fig. 8 F) is associated with myofilament Ca^2+^ sensitivity; the rate constant of force development (Fig. 8, B and G) reflects XB turnover rate; the duration (Fig. 8, C and H) and rate constant (Fig. 8, D and I) of the slow phase of relaxation reflect XB detachment kinetics; and the rate constant of the fast phase of relaxation (Fig. 8, E and J) is influenced by sarcomere compliances and inter-sarcomere dynamics. There are no differences among groups for any of these parameters, suggesting that the increased diastolic XB stress in male HFpEF-like is not attributable to alterations in myofilament XB kinetics.

## Discussion

We quantified the relative contributions of passive sarcomere stiffness, the microtubule network, and diastolic XB activity to total diastolic stress in intact cardiomyocytes isolated from two-hit mice, a cardiometabolic HFpEF-like model, to identify potential therapeutic targets for reducing cardiomyocyte diastolic stiffness in both sexes. Our results show that diastolic XBs are important contributors to diastolic stress, and they are significantly increased in male HFpEF-like mice compared with male-ctrl, whereas no increase is observed in female HFpEF-like mice. The ratio of XB-to-total diastolic stress is highest at shorter SLs and decreases as SL increases, indicating that diastolic XBs are the primary contributor at short SLs, whereas non-XB mechanisms dominate at longer SLs. At SLs ≤2.15 μm, microtubules do not directly contribute to diastolic stress through their mechanical stiffness; instead, they contribute to diastolic stress by enhancing diastolic XB activity, particularly in male mice. There is increased passive sarcomere stress in cardiomyocytes of both male and female HFpEF-like mice. Altered Ca^2+^ handling is observed in male HFpEF-like, evidenced by increased Ca^2+^ transient amplitude, but not in female HFpEF-like mice. This Ca^2+^ change parallels the increase in diastolic XB stress in male HFpEF-like, while no significant changes in myofilament XB kinetics are detected.

The contributors to diastolic stress differ between males and females, supporting the importance of sex-specific therapeutics in HFpEF. In males, both increased sarcomere passive stiffness and diastolic XB activity represent therapeutic targets, whereas, in females, diastolic stiffness is mainly driven by elevated passive sarcomere stiffness. However, it is possible that therapeutic targets in females extend beyond cardiomyocyte-intrinsic mechanisms and include factors such as extracellular matrix remodeling, ventricular geometry, ventricular-arterial coupling, and coronary endothelial dysfunction.

### Potential mechanisms underlying increased diastolic XBs

Diastolic XBs act as a diastolic resting tone across the physiological SL range. The findings of this study are consistent with previous works from our group (King et al., 2011; Methawasin et al., 2014) and others (Sequeira et al., 2015a; Sequeira et al., 2015b). The magnitude of diastolic XBs is determined by (1) the level of cytoplasmic Ca^2+^ (Sequeira et al., 2015a; Selby et al., 2011; Runte et al., 2017), (2) the energetic status, specifically the level of ADP (Donaldson et al., 2012; Sequeira et al., 2015a; Sequeira et al., 2015b), and (3) myofilament intrinsic properties (Janssen, 2019; Chung et al., 2016; Rao et al., 2014; Toepfer et al., 2016; Rosas et al., 2015; Martins et al., 2015; Locher et al., 2011; Lee et al., 2010; Lee et al., 2013), as well as the interactions among these factors. Elevated myofilament Ca^2+^ sensitivity, enhanced length-dependent activation, and delayed XB detachment can all increase diastolic XB formation (Janssen, 2019; Chung et al., 2016; Donaldson et al., 2012).

Recently, the positioning of myosin heads between the disordered-relaxed (DRX) and super-relaxed (SRX) states has become an important topic in the myofilament field. Both DRX and SRX represent relaxed states of myosin, with DRX heads position closer to actin and available for force generation (Schmid and Toepfer, 2021; Nag and Trivedi, 2021; Stewart et al., 2010), whereas SRX heads interact with one another and fold back onto the thick filament backbone (McNamara et al., 2015; Toepfer et al., 2020; Hooijman et al., 2011; McNamara et al., 2017; Schmid and Toepfer, 2021). A shift in myosin head populations toward the DRX state increases the likelihood of recruitment into force-generating XBs. An increased DRX population has been linked to hypercontractility and diastolic dysfunction in HCM (McNamara et al., 2015; Toepfer et al., 2020; Schmid and Toepfer, 2021). However, knowledge of SRX/DRX distribution in HFpEF remains limited. Given that HCM and HFpEF share key diastolic features, including impaired relaxation and elevated diastolic XBs (Selby et al., 2011; Runte et al., 2017; Donaldson et al., 2012), depopulation of SRX myosin (e.g., myosin recruitment) may likewise occur in some sub phenotype of HFpEF.

An increase in diastolic XBs may indicate a potential shift of myosin heads to DRX states. Although in this study, we did not observe changes in XB kinetics or myofilament Ca^2+^ sensitivity (Fig. 8, A–J), suggesting that alterations in the SRX/DRX ratio are unlikely; however, such changes cannot be definitively excluded. Direct measurement of SRX/DRX populations requires specialized techniques, such as X-ray diffraction (Ma et al., 2022; Ma et al., 2023; Jani et al., 2024b) or the multiple turnover assays with the fluorescent form of ATP (Mohran et al., 2024; Steczina et al., 2025; Pilagov et al., 2025), which are not included in this study.

Myofilament intrinsic properties vary with isoform composition and PTMs and are also influenced by experimental conditions such as lattice spacing and temperature (Harrison and Bers, 1989; Harrison and Bers, 1990a; Harrison and Bers, 1990b). Diastolic XBs are typically absent in permeabilized cardiomyocytes or myocardial preparations, as these lack an intact plasma membrane, exhibit expanded lattice spacing, and are generally studied at room temperature or below (Farman et al., 2006). The presence of diastolic XBs underscores the importance of intact preparations, which preserve the plasma membrane, maintain physiological lattice spacing, and are typically studied at physiological temperature.

Dysregulation of Ca^2+^ handling is influenced by comorbidities and sex and has been reported in cardiomyocytes from both human patients and animal models of HFpEF (Hegyi et al., 2026; Mira Hernandez et al., 2025; Sequeira et al., 2025; Frisk et al., 2021). In the present study, we did not observe elevated diastolic Ca^2+^ levels (Fig. 7 A); however, we detected an increased Ca^2+^ transient amplitude in male HFpEF-like (Fig. 7 B), along with a significant main effect of HFpEF on a trend toward rapid Ca^2+^ reuptake (Fig. 7 D). Although Ca^2+^ transient amplitude primarily reflects sarcoplasmic reticulum Ca^2+^ release during systole and may not directly explain the increased diastolic XBs observed here, it suggests alterations in Ca^2+^ handling in male HFpEF-like. Such changes could arise from modifications in Ca^2+^-handling proteins or heart rate. Because intact cardiomyocytes in this study were paced at 4 Hz, heart rate differences can be excluded, making altered Ca^2+^-handling proteins the more likely explanation.

In addition to myofilament properties and Ca^2+^ handling, energetic deficits under pathological cardiac conditions may also contribute to elevated diastolic XBs by impairing XB detachment, particularly through increased cytoplasmic ADP levels (Donaldson et al., 2012; Sequeira et al., 2015a; Sequeira et al., 2015b). Previous studies have shown that elevated ADP can induce diastolic dysfunction even when ATP levels are normal and that energetic deficiency can impair diastolic function before affecting systolic function (Tian et al., 1997). ADP accumulation can increase myofilament Ca^2+^ sensitivity and trigger myofilament contraction even in the absence of Ca^2+^. Elevated myocardial ADP promotes XB recruitment and slows XB detachment, thereby increasing diastolic XB formation (Sequeira et al., 2015a; Sequeira et al., 2015b). Energetic deficits may contribute to the increased diastolic XBs observed in intact cardiomyocytes (Fig. 3 A) despite normal XB kinetic parameters in myofibrils (Fig. 8, A–J).

### Microtubules contribute to diastolic stress through the enhancement of diastolic XB activity

We did not detect a statistically significant reduction in diastolic stress in cardiomyocytes with colchicine-induced microtubule depolymerization (Fig. 4). However, we observed that microtubules significantly modulate the magnitude of diastolic XB stress in male mice (Fig. 5, A and B). Beyond their mechanical role, microtubules perform multiple critical functions, including intracellular transport of proteins and other cargo, organization of subcellular domains and intercalated discs, and maintenance of dyad structure composed of T-tubules and sarcoplasmic reticulum ryanodine receptors (Chen et al., 2018; Caporizzo and Prosser, 2022). The reduction in diastolic XB activity following microtubule depolymerization (Fig. 5, A and B) may therefore be mediated by microtubule-dependent effects on Ca^2+^ handling.

The absence of a statistically significant reduction in diastolic stress following colchicine treatment may be partially explained by the predominant contribution of microtubules to viscous rather than elastic components of cellular stress (Loescher et al., 2023; Robison et al., 2016). Viscous stress is dependent on stretch velocity and is more pronounced at longer SLs (Chung et al., 2011a; Chung et al., 2011b). Because, in this study, the cells were stretched only to an SL of 2.15–2.2 µm to avoid compromising cell attachment to the glass rods, the mechanical contribution of microtubular stiffness may not have been fully captured under these experimental conditions.

### Possible causes of increased passive sarcomere stiffness

The increased passive sarcomere stress observed in this study (Fig. 6, A and B) is likely due to alterations in titin, which is the primary regulator of cardiomyocyte passive sarcomere properties (Chung and Granzier, 2011; Granzier and Irving, 1995; Loescher et al., 2023). Titin stiffness can be regulated through shifts in isoform expression and PTMs (Chung et al., 2013; Hidalgo and Granzier, 2013; LeWinter and Granzier, 2010; Loescher et al., 2022; Granzier and Labeit, 2025). Aberrant titin PTMs and increased sarcomere-based stiffness have been consistently reported in multiple HFpEF studies in both human patients and animal models (Borbely et al, 2005; Bishu et al, 2011; Van Heerebeek et al, 2012b; Hamdani et al, 2013a, 2013b, 2017; Zile et al, 2015; Kovacs et al, 2016; Hopf et al, 2018; Jeong et al, 2018; Bai et al, 2019; Methawasin et al, 2020; Abdellatif et al, 2021; Kolijn et al, 2021; Soetkamp et al, 2021; Koser et al, 2022; Lin et al, 2022).

In addition to titin, recent work by Loescher et al. has shown that actin also contributes to cardiomyocyte viscoelastic properties, both through interactions with titin (Yamasaki et al., 2001; Chung et al., 2011a) and via its intrinsic viscoelasticity, independent of titin (Loescher et al., 2023). This finding aligns with proteomic analyses in HFpEF-like rat models and human HFpEF myocardium, which revealed significant alterations in actin filament-binding, actin-organizing proteins, and actin cytoskeleton structures (Soetkamp et al., 2021; Jani et al., 2025). Together, these suggest that actin may play a more prominent role in regulating sarcomere passive properties than previously recognized and warrant further investigation. Although HFpEF-like mice of both sexes exhibit increased passive sarcomere stiffness, it remains possible that the underlying sarcomeric protein alterations differ between males and females. This question will be addressed in our follow-up studies.

### Phenotypic variability of the two-hit model among different studies

Although the two-hit model is a well-established model of cardiometabolic HFpEF, it exhibits high variability in phenotype severity. As we discussed in our previous work (Methawasin et al., 2025b), we observed seasonal variations: mice subjected to the two-hit regimen in summer gained more weight and developed a stronger HFpEF-like phenotype compared with those set up in winter. Phenotype expression, particularly in male mice, was also influenced by housing conditions, as single-housed mice tended to gain more weight and develop a more pronounced phenotype. We also observed variability across facilities as mice subjected to the two-hit regimen developed a HFpEF-like phenotype at the University of Arizona, whereas at the University of Missouri, the phenotype was less pronounced, with less weight gain and a smaller increase in blood pressure. Notably, we were unable to reproduce the increased lung water phenotype as originally reported (Schiattarella et al., 2019). These variabilities may contribute to discrepancies among studies using the two-hit model. For example, previous reports (Jani et al., 2024a; Fenwick et al., 2024) have described delayed XB kinetics without an increase in sarcomere passive stress in two-hit mice, whereas both our prior work (Methawasin et al., 2025a) and the present study demonstrate enhanced passive sarcomere stress without detectable changes in XB kinetics. We speculate that these differences likely reflect phenotypic variability.

However, a key advantage of the two-hit regimen is that it is an acquired model, as the metabolic conditions develop in adulthood rather than being a congenital disorder, similar to the natural progression of HFpEF in humans. Additionally, the two-hit mice do not progress to heart failure with reduced ejection fraction, as is also the case in human HFpEF. Modifications of the two-hit regimen, such as combining a high-fat diet with angiotensin II infusion (Withaar et al., 2023), or using db/db mice with aldosterone supplementation (Hegyi et al., 2022; Mira Hernandez et al., 2025; Hegyi et al., 2026), may serve as useful alternatives. However, the resulting phenotype and underlying molecular mechanisms may differ from those of the original two-hit model (high-fat diet plus L-NAME). In fact, this diversity of animal models is not a limitation, as HFpEF is a heterogeneous syndrome with multiple phenotypes driven by different comorbidities. Our group also plans to extend this work to other HFpEF sub-phenotypes, such as chronic kidney disease–associated HFpEF and cardiovascular-kidney-metabolic syndrome.

### Study limitations

In addition to inhibiting actomyosin XB cycling by slowing phosphate release and stabilizing the myosin-ADP-Pi state of the ATPase cycle (Herrmann et al., 1992), BDM is known to exert several non-XB effects. BDM suppresses both the slow inward Ca^2+^ current and transient outward current, resulting in alterations in cardiac action potential duration and diminished loading of sarcoplasmic reticulum (Gwathmey et al., 1991; Coulombe et al., 1990). Furthermore, BDM has been reported to possess protein phosphatase activity, which may influence the phosphorylation status of contractile and Ca^2+^ regulatory proteins (Stapleton et al., 1998). Therefore, the effects of BDM are not exclusively attributed to inhibition of XB cycling.

A more selective XB inhibitor, blebbistatin, inhibits XB cycling by binding to a hydrophobic pocket within the myosin motor domain and stabilizing a low actin-affinity state (Kovács et al., 2004). In our previous studies (King et al., 2011), measurements of diastolic XB extent were comparable between BDM- and blebbistatin-treated cells, suggesting that BDM did not affect the assessment of diastolic XB activity. Nevertheless, the potential non-XB effects of BDM should be considered when interpreting the results.

The present study focuses on identifying the relative contributions of different determinants of diastolic dysfunction in the HFpEF-like model. While our findings provide physiological insights and establish associations among these contributors, the study did not cover the mechanistic pathways linking individual observations. Future studies are needed to investigate the molecular and cellular mechanisms underlying these findings, as well as their sex-specific differences.

### Clinical implication

Preclinical studies of myofilament mechanobiology in HFpEF are essential, as understanding how each mechanical element contributes to diastolic dysfunction can guide the identification of the most promising therapeutic targets, which is critical for developing personalized HFpEF therapies (Mishra and Kass, 2021; Shah et al., 2020). For example, patients with a fibrotic phenotype may benefit from anti-fibrosis treatments (Lewis and Miller, 2017); those with increased sarcomere passive stiffness could benefit from strategies that upregulate compliant titin isoforms (Radke et al., 2021; Methawasin et al., 2016; Methawasin et al., 2025a); patients with increased microtubular proliferation may respond to drugs targeting microtubules (Robison et al., 2016; Chen et al., 2018; Eaton et al., 2024; Pietsch et al., 2024); and HFpEF dominated by diastolic XBs or altered DRX/SRX states may potentially benefit from myosin inhibitors (Gollapudi et al., 2021), among other phenotype-specific approaches.

### Conclusions

Diastolic stress is increased in cardiomyocytes from the two-hit mouse model of cardiometabolic HFpEF. In female mice, this increase is primarily driven by elevated passive sarcomere stress. Whereas in males, it is attributable to both increased passive sarcomere stress and enhanced diastolic XB formation, which is likely due to pathological remodeling in Ca^2+^ handling. The microtubular network contributes to diastolic stress through enhancing the magnitude of diastolic XBs. These findings suggest that cardiomyocytes represent an important therapeutic target in HFpEF; however, the contributors to diastolic stress differ between males and females, supporting the importance of sex-specific therapeutics in HFpEF.

## Supplementary Material

Review History

Table S1 shows echocardiographic parameters.

## Data Availability

The datasets supporting this study are available in the Harvard Dataverse repository: https://dataverse.harvard.edu/dataverse/202614028R1.
