# Supplementary material for: Diastolic contributors in cardiomyocytes of a cardiometabolic HFpEF-like mouse model
Source: J Gen Physiol. 2026 Aug 25;158(6):e202614028. doi: 10.1085/jgp.202614028 (PMC13505484; doi:10.1085/jgp.202614028)
Supplement: Supplementary file 1 [file jgp_202614028_review_history.pdf]

Diastolic contributors in cardiomyocytes of a cardiometabolic HFpEF-like mouse model.

Arooj Shahid, Timothy McMillen, Samuel Daughtery, Anna Burns, Marloes van den Berg, Michael Regnier, Henk Granzier, and Mei Methawasin

Corresponding Author(s): Mei Methawasin, University of Missouri

Review Timeline:

Submission Date:	April 11, 2026
Editorial Decision:	May 6, 2026
Revision Received:	July 1, 2026
Editorial Decision:	July 27, 2026
Revision Received:	August 2, 2026

Editor: Olaf Andersen

Transaction Report:

(Note: With the exception of the correction of typographical or spelling errors that could be a source of ambiguity, letters and reports are not edited. The original formatting of letters and referee reports may not be reflected in this compilation.)

DOI: https://doi.org/10.1085/jgp.202614028

May 6, 2026

Dr. Mei Methawasin
University of Missouri
Medical Pharmacology and Physiology
1 Hospital Dr
MA415 MSB
Columbia, MO 65212

Re: 202614028

Dear Dr. Methawasin,

Thank you for submitting your manuscript, entitled "Sex differences in diastolic contributors in cardiomyocytes of the cardiometabolic HFpEF mouse model." to JGP. Your manuscript has now been seen by 2 reviewers, whose comments are appended below. You will see that the reviewers have raised important concerns that should be addressed prior to further consideration of the manuscript at JGP. In particular, please view the editor's summary directly below.

Editor's Summary
Based on the reviewers' comments and my own reading, you need to undertake an extensive revision, in which you address the letter and spirit of the reviewers concerns. In addition to the reviewers' comments, I wonder whether you should revise the title, changing "of the cardiometabolic" to "of a cardiometabolic", and I was struck by the different conclusions you reached based on the results for males and females, why are there no therapeutic implications of the results on female cardiomyocytes? I also find that you should acknowledge that 20 mM 2,3-BDM has numerous effects that do not involve crossbridges; how might that impact on your conclusions. With respect to figures (comment by reviewer 1), I find panel panel C in Figure 5 to be uninterpretable, and Figure 7 to be too crowded.

We would be pleased to receive a suitably revised manuscript that addresses/resolves these concerns, which will be re-reviewed, most likely by some or all of the original referees. In addition, please do not hesitate to contact me (via the editorial office) if you feel that a discussion of the reviewers' and editors' comments would be helpful.

Please submit your revised manuscript via the link below along with a point-by-point letter that details your responses to the editor's summary and reviewers' comments, as well as a copy of the text with alterations highlighted (boldfaced or underlined). If the article is eventually accepted, it would include a 'revised date' as well as submitted and accepted dates. If we do not receive the revised manuscript within one year, we will regard the article as having been withdrawn. We would be willing to receive a revision of the manuscript at a later time, but the manuscript will then be treated as a new submission, with a new manuscript number.

Please pay particular attention to recent changes to our instructions to authors in the following sections: Data presentation, Blinding and randomization and Statistical analysis, under Materials and Methods, as shown here: https://rupress.org/jgp/pages/submission-guidelines#prepare. Re-review will be contingent on inclusion of the required information (including for data added during revision) and demonstration of the experimental reproducibility of the results. Also, to improve the reproducibility of published content, we have partnered with SciScore. Authors are prompted in eJP to copy and paste the Materials and Methods section of their manuscript for a SciScore assessment when submitting their revised manuscript. Authors are encouraged (not required) to further revise their Materials and Methods if the SciScore is below 4. More information can be found here: https://rupress.org/jgp/pages/submission-guidelines#sciscore

Please note, JGP requires authors to submit Source Data used to generate figures containing gels and Western blots with all revised manuscripts (when applicable). This Source Data consists of fully uncropped and unprocessed images for each gel/blot displayed in the main and supplemental figures. If your paper includes cropped gel and/or blot images, please be sure to provide one Source Data file for each figure that contains gels and/or blots along with your revised manuscript files. File names for Source Data figures should be alphanumeric without any spaces or special characters (i.e., SourceDataF#, where F# refers to the associated main figure number or SourceDataFS# for those associated with Supplementary figures). The lanes of the gels/blots should be labeled as they are in the associated figure, the place where cropping was applied should be marked (with a box), and molecular weight/size standards should be labeled wherever possible.
Source Data files will be made available to reviewers during evaluation of revised manuscripts and, if your paper is eventually published in JGP, the files will be directly linked to specific figures in the published article.

Source Data Figures should be provided as individual PDF files (one file per figure). Authors should endeavor to retain a minimum resolution of 300 dpi or pixels per inch. Please review our instructions for export from Photoshop, Illustrator, and PowerPoint here: https://rupress.org/jgp/pages/submission-guidelines#revised

When revising your manuscript, please be sure it is a double-spaced MS Word file and that it includes editable tables, if appropriate.

Please submit your revised manuscript via this link:
Link Not Available

Thank you for the opportunity to consider your manuscript.

Sincerely,

Olaf S. Andersen, M.D.
On behalf of Journal of General Physiology

Journal of General Physiology's mission is to publish mechanistic and quantitative molecular and cellular physiology of the highest quality; to provide a best-in-class author experience; and to nurture future generations of independent researchers.

Reviewer #1 (Comments to the Authors):

Shahid et al., JGP 2026
In this report, the authors perform challenging, intact, single-myocyte mechanical measurements to assess the relative contributors of various elements to diastolic stress in a cardiometabolic murine model of HFpEF, and to assess their contribution by gender.
The introduction is both thorough and clearly written, as is the methodology. The experimental design is clearly explained and the study is well justified. Measurements appear sufficiently powered, an impressive feat with challenging and low throughput approaches. There are a number of polished aspects to the design, execution, and presentation. My concerns largely surround aspects of data presentation and interpretation.
1. I have some confusion in interpreting the single cell stretch approach and observations that I hope the authors can clarify.
a. Are both glass rods equally compliant, and what is the nature of the contraction? If the rods were both rigid, one might anticipate relatively isometric contractions, yet sarcomere length decreases by >10% upon stimulation in Figure 1, suggesting more of an auxotonic contraction?
b. Active force appears rather small, then upon stretch the passive force appears of unexpectedly high magnitude; the passive force appears ~5x higher than active force, which does not seem to match the cardiac length tension relationship (even accounting for being on different points of the Starling Curve).
c. Further, the piezo step is rather large - 50um step, with 80um distance between the rods; this would mean the strain should be ~60%, yet SL increases 15-20%. Can the authors thus explain where the slip in the system occurs, and whether this slip is fixed or time varying, as this could influence test-re-test variability, which is an integral part of their experimental design.
2. Please clarify the concentration of BDM used (it says 20mM in methods, 20uM in figure legend). While I agree with the authors interpretation of informing on XB stress, as BDM can have multiple on and off target effects (especially if at 20mM), I would label the actual figure panels in 2A-D as before and after BDM, and then the interpretation/breakdown in Fig 2E (cross-bridge stress vs. non-cross bridge stress) is reasonable.
3. Regardless, the change in diastolic stress as a function of SL with and without BDM in Figure 2 is presented transparently. In contrast, the way the colchicine data is presented is more convoluted, and the interpretation is speculative (and potentially misleading). The authors should present diastolic stress (from the initial stretch ramp) as a function of SL with and without colchicine treatment in the same transparent fashion. Then, they could report the BDM-dependent component to look at potential interdependencies. Instead, the authors present only the diastolic stress remaining after both colchicine and BDM treatment (second stretch, labeled as "non-XB stress"). The lack of effect on this parameter is then interpreted as "the microtubules do not contribute measurably to diastolic stress". Yet two figures later, the authors show that colchicine reduces the magnitude of "XB stress" (the difference in stress between the first and second stretch after BDM). If I understand the author's definitions, this implies there was some modest drop in the diastolic stress (first stretch) upon colchicine treatment (in males), and then less of difference upon the second stretch after BDM treatment. That is an interesting piece of data supporting microtubule-myofilament mechanical interdependency, but it should be presented and interpreted more appropriately. An interpretation of microtubules do not contribute to diastolic stress is misleading - the authors data in fact supports that colchicine treatment reduced diastolic stress in some conditions - but certainly this contribution could be influenced by residual actomyosin tension. One cannot only measure the impact of a mechanical element after removing another element from a mechanical system (especially a complex one where those elements, here microtubules and myofilaments, are known to interact). A simplified equivalent would be to remove all cables from a strut and cable structure - the system would collapse, and subsequent measurements of the strut's contribution to the mechanical properties of the system would be irrelevant. The results for this section should thus be revised and clarified, and discussion/speculation around potential interpretations of these observations restricted to the discussion.
4. The proposed link between elevated peak systolic calcium levels and diastolic XB contribution in HFpEF males also feels insufficiently justified, particularly given that the authors measure no change in diastolic calcium levels or myofilament calcium

sensitivity. The same goes for proposed links between microtubule changes and changes to EC coupling/calcium levels, particularly without any measurements of ECC +/- colchicine. Such speculation should be moved at least out of the results section, and more carefully considered in the discussion.
Minor:
1. Please consider adding more contrast between groups for figure presentation (e.g. color the data points, bolder lines, etc) - sometimes it is difficult to discriminate the groups.
2. Figure 2E seems to be missing x-axis labels to define groups.

Reviewer #2 (Comments to the Authors):

This is an interesting work. Overall the findings are important for the field however, I do have a few concerns to address:

MAJOR
Isn't it reductionist to restrict diastolic stiffness to three components. Authors should consider other contributors.

Is it a real model of human HFpEF or diastolic dysfunction? Authors should be cautious

Age is not addressed at all (rationale, design, results...). This is important especially considering the model used.

MINOR
Intro:
1st paragraph: 'etc' is not very scientific and should be refined properly.
3rd paragraph: Overall, it is not easy to read. Also, please mention human data on microtubules.
4th paragraph: The rationale for studying sex differences is not presented in depth, and the hypothesis is not backed up by any references or justification.
5th-6th-7th paragraph: The part on the 2-hit model is difficult to read with lots of repetition and should be put in the context of other models.

Methods:
A table summarising cardiac function in the various groups is needed.

Results:
Choice of SLs in general is unclear.
Link between the individual findings are not clear as approaches are descriptive rather than mechanisitic.

Editor's comments

We sincerely thank the Editor for evaluating our work, the opportunity to revise the manuscript, and the constructive comments and suggestions.

1) *In addition to the reviewers' comments, I wonder whether you should revise the title, changing "of the cardiometabolic" to "of a cardiometabolic".*

Response. According to the Editor's and Reviewer 2' s comments, we have revised the title from "Sex Differences in Diastolic Contributors in Cardiomyocytes of the Cardiometabolic HFpEF Mouse Model" to "Diastolic Contributors in Cardiomyocytes of a Cardiometabolic HFpEF-like Mouse Model".

2) *I was struck by the different conclusions you reached based on the results for males and females. Why are there no therapeutic implications of the results on female cardiomyocytes?*

Response. "In males, both increased sarcomere passive stiffness and diastolic XB activity represent therapeutic targets, whereas, in females, diastolic stiffness is mainly driven by elevated passive sarcomere stiffness. However, it is possible that therapeutic targets in females extend beyond cardiomyocyte-intrinsic mechanisms and include factors such as extracellular matrix remodeling, ventricular geometry, ventricular-arterial coupling, and coronary endothelial dysfunction."

We have revised the Discussion section to incorporate the statements above. Please refer to lines 399-402 of the revised manuscript.

3) *I also find that you should acknowledge that 20 mM 2,3-BDM has numerous effects that do not involve crossbridges; how might that impact on your conclusions.*

Response. In response to the comments from the editor and Reviewer 2 (Comment 2), we have revised the Discussion section to acknowledge the non-crossbridge effects of BDM as a limitation of the study. Please refer to lines 536-549 of the revised manuscript.

"In addition to inhibiting actomyosin XB cycling by slowing phosphate release and stabilizing the Myosin-ADP-Pi state of the ATPase cycle (Herrmann et al., 1992), BDM is known to exert several non-XB effects. BDM suppresses both the slow inward Ca^{2+} current and transient outward current, resulting in alterations in cardiac action potential duration, and diminished loading of sarcoplasmic reticulum(Gwathmey et al., 1991, Coulombe et al., 1990). Furthermore, BDM has been reported

to possess protein phosphatase activity, which may influence the phosphorylation status of contractile and Ca2+ regulatory proteins(Stapleton et al., 1998). Therefore, the effects of BDM are not exclusively attributed to inhibition of XB cycling.

A more selective XB inhibitor, blebbistatin, inhibits XB cycling by binding to a hydrophobic pocket within the myosin motor domain and stabilizing a low actin-affinity state (Kovács et al., 2004). In our previous studies (King et al., 2011), measurements of diastolic XB extent were comparable between BDM- and blebbistatin-treated cells, suggesting that BDM did not affect the assessment of diastolic XB activity. Nevertheless, the potential non-XB effects of BDM should be considered when interpreting the results."

4) *With respect to figures (comment by reviewer 1), I find panel C in Figure 5 to be uninterpretable, and Figure 7 to be too crowded.*

Response. We have revised the original Figure 5C (revised Figure 6C) by separating the data into four individual panels representing the male-control, male HFpEF-like, female-control, and female HFpEF-like groups. Additionally, the original Figure 7 has been divided into revised Figures 7 and 8 to improve clarity and presentation.

Reviewer#1:

Shahid et al., JGP 2026

In this report, the authors perform challenging, intact, single-myocyte mechanical measurements to assess the relative contributors of various elements to diastolic stress in a cardiometabolic murine model of HFpEF, and to assess their contribution by gender. The introduction is both thorough and clearly written, as is the methodology. The experimental design is clearly explained and the study is well justified. Measurements appear sufficiently powered, an impressive feat with challenging and low throughput approaches. There are a number of polished aspects to the design, execution, and presentation. My concerns largely surround aspects of data presentation and interpretation.

Response. We thank the reviewer for the careful evaluation of our manuscript and for the insightful and constructive comments. We appreciate the reviewer's recognition of the effort involved in performing these technically challenging intact single-cardiomyocyte mechanical measurements.

We also appreciate the reviewer's concerns regarding aspects of data presentation and interpretation. In response, we have carefully considered each comment and revised the manuscript accordingly to improve clarity, strengthen the interpretation of the findings, and better present the data. Detailed responses to each point are provided below.

1.) I have some confusion in interpreting the single-cell stretch approach and observations that I hope the authors can clarify.

a. Are both glass rods equally compliant, and what is the nature of the contraction? If the rods were both rigid, one might anticipate relatively isometric contractions, yet sarcomere length decreases by >10% upon stimulation in Figure 1, suggesting more of an auxotonic contraction?

Response. Although we did not directly measure the stiffness of individual glass rods, they are expected to have comparable stiffness and to be much stiffer than the carbon fiber system used in our previous studies (PMIDs: 21187335, 24599837), in which cardiomyocytes contracted under auxotonic loading conditions. The myostretcher system was designed to approximate isometric contraction. However, it is not strictly isometric due to the system compliance, primarily resulting from deformation of the glass needles and MyoTak (the adhesive). Nevertheless, the contractions remained distinct from auxotonic conditions. Although the cell was attached at both ends and the overall cell length remained relatively constant during contraction, sarcomere shortening was still observed.

b. Active force appears rather small, then upon stretch the passive force appears of unexpectedly high magnitude; the passive force appears ~5x higher than active force, which does not seem to match the cardiac length tension relationship (even accounting for being on different points of the Starling Curve).

Response. The diastolic: systolic force ratio is high because systolic force is measured at the lower end on the Frank-Starling curve (SL ~1.6 μm), due to the system compliance, while the diastolic force is measured at longer SL (≥ 2.15 μm). In addition, we anticipate that elevated diastolic force is also due to increased diastolic crossbridge stress, particularly under pacing conditions at 4 Hz.

The representative tracings shown in the original Figure 1 were obtained from a male HFpEF-like cardiomyocyte and therefore exhibited relatively high diastolic stress. We selected this example cell because it provided a particularly clean sarcomere-length tracing, which we believed would facilitate interpretation for the reader. However, we agree with the reviewer that this cell may not

be the most representative example. Therefore, we have replaced the original tracings with ones from a control cardiomyocyte that shows a lower diastolic-to-systolic force ratio. Although the sarcomere length tracing in the revised example cell is not as clean as in the original figure, we believe it provides a more representative illustration of the typical experimental recordings.

c. Further, the piezo step is rather large - 50um step, with 80um distance between the rods; this would mean the strain should be ~60%, yet SL increases 15-20%. Can the authors thus explain where the slip in the system occurs, and whether this slip is fixed or time varying, as this could influence test-re-test variability, which is an integral part of their experimental design.

<u>Response.</u> Because of the system compliance, sarcomere stretch does not precisely match the displacement imposed by the piezo, with sarcomere stretch being smaller than the applied displacement. Therefore, we relied primarily on the measured sarcomere lengths. To avoid potential confusion, we have revised the y-axis in Figure 1B, Piezo position tracing, from piezo movement (μm) to length profile.

To verify proper cell attachment, a four-stretch protocol was applied. For each cell, a total of four trapezoid stretches were performed, using the same stretch amplitude, as illustrated in an example figure below. The first stretch was used to ensure that the sarcomere length reached ≥ 2.15 μm and to verify secure cell attachment. The second stretch was used to obtain force before BDM treatment. BDM was then introduced into the perfusion solution, and the third stretch was performed to obtain force after BDM treatment. Finally, the fourth stretch was performed to confirm that cell attachment remained stable throughout the experiment. The stress reduction after BDM treatment represented the extent of diastolic XB stress. A reduction in stress during the fourth stretch compared with the third stretch was considered indicative of loosening of the attachment, and those cells were excluded from analysis.

We have revised the Methods section to include the four-stretch protocol described above (please refer to lines 203-212 of the revised manuscript.),and added a representative example of Four-stretch protocol in Supplementary Figure 1.

Example tracings of the four-stretch protocol

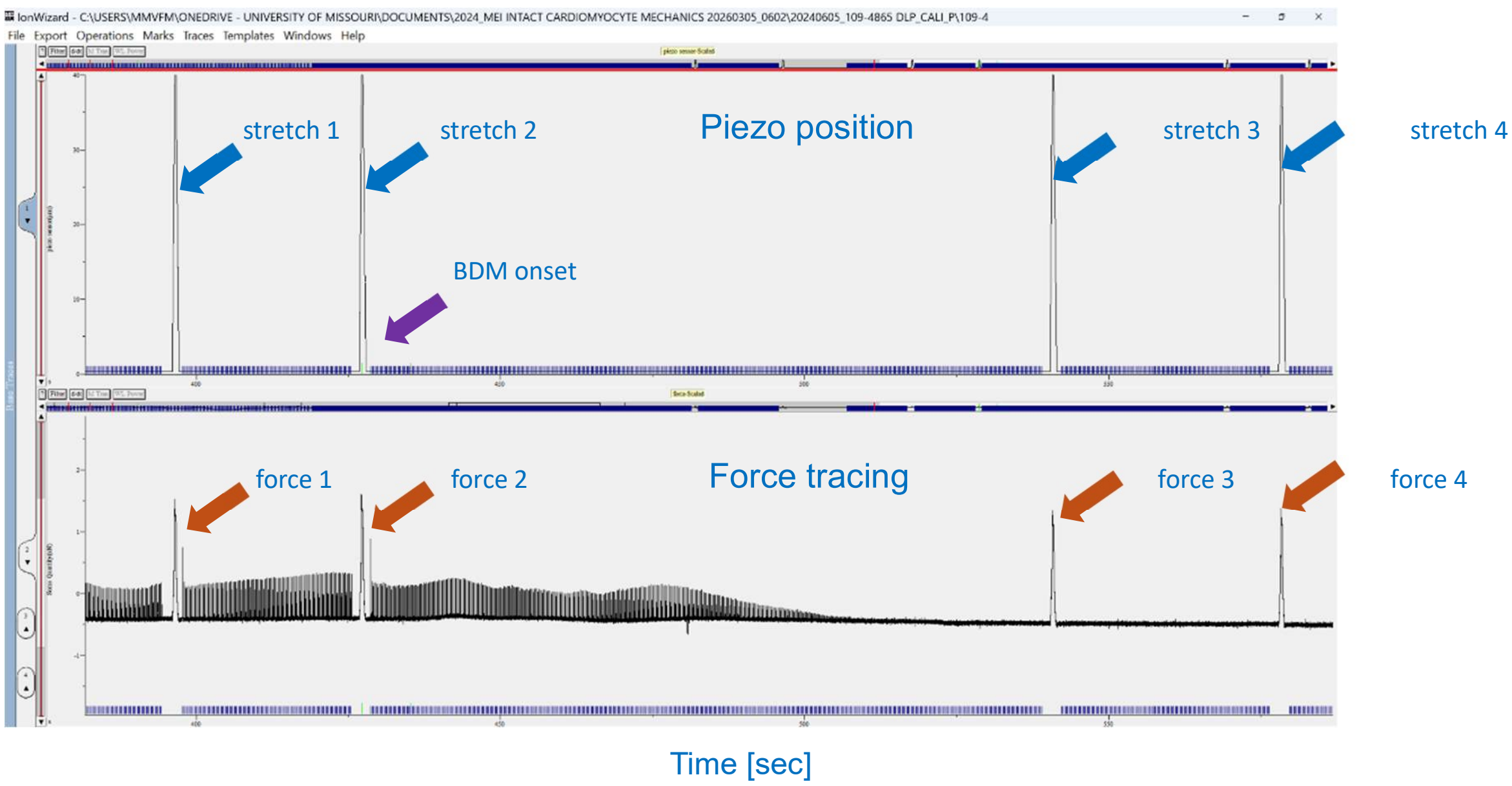

2.) Please clarify the concentration of BDM used (it says 20mM in methods, 20uM in figure legend). While I agree with the authors interpretation of informing on XB stress, as BDM can have multiple on and off target effects (especially if at 20mM), I would label the actual figure panels in 2A-D as before and after BDM, and then the interpretation/breakdown in Fig 2E (cross-bridge stress vs. non-cross bridge stress) is reasonable.

<u>Response.</u> We apologize for the typo. The correct concentration of BDM used in this study is 20 mM. We have corrected this in the Figure 1 legend. In addition, we have revised the labels in Figure 2A-D by replacing “XB inhibitor” with “BDM”.

In response to the comment regarding the non-XB effect of BDM by reviewer 1 and the editor, we have revised the Discussion section to acknowledge the non-cross-bridge effects of BDM as a limitation of the study. Please refer to the revised manuscript lines 536-549, or editor’s comment 3.

3.) Regardless, the change in diastolic stress as a function of SL with and without BDM in Figure 2 is presented transparently. In contrast, the way the colchicine data is presented is more convoluted, and the interpretation is speculative (and potentially misleading).

The authors should present diastolic stress (from the initial stretch ramp) as a function of SL with and without colchicine treatment in the same transparent fashion. Then, they could report the BDM-dependent component to look at potential interdependencies.

Instead, the authors present only the diastolic stress remaining after both colchicine and BDM treatment (second stretch, labeled as "non-XB stress"). The lack of effect on this parameter is then interpreted as "the microtubules do not contribute measurably to diastolic stress". Yet two figures later, the authors show that colchicine reduces the magnitude of "XB stress" (the difference in stress between the first and second stretch after BDM). If I understand the author's definitions, this implies there was some modest drop in the diastolic stress (first stretch) upon colchicine treatment (in males), and then less of difference upon the second stretch after BDM treatment. That is an interesting piece of data supporting microtubule-myofilament mechanical interdependency, but it should be presented and interpreted more appropriately.

An interpretation of microtubules do not contribute to diastolic stress is misleading - the authors data in fact supports that colchicine treatment reduced diastolic stress in some conditions - but certainly this contribution could be influenced by residual actomyosin tension.

One cannot only measure the impact of a mechanical element after removing another element from a mechanical system (especially a complex one where those elements, here microtubules and myofilaments, are known to interact). A simplified equivalent would be to remove all cables from a strut and cable structure - the system would collapse, and subsequent measurements of the strut's contribution to the mechanical properties of the system would be irrelevant.

The results for this section should thus be revised and clarified, and discussion/speculation around potential interpretations of these observations restricted to the discussion.

Response. We thank the reviewer for this insightful comment. We have revised Figure 4 to include diastolic stress - SL relations from colchicine- and DMSO-treated cells, both before and after BDM treatment. Although no statistically significant effect of colchicine on diastolic stress was observed in any group, the male HFpEF-like group showed a trend toward reduced diastolic stress in colchicine-treated cells prior to BDM treatment (revised Figure 4B). However, statistical significance was detected when XB stress was analyzed (revised Figure 5), but not when diastolic stress was used for comparison. These findings suggest that microtubules contribute to diastolic stress by enhancing diastolic XB activity, particularly in male mice. Please refer to lines 319-328 of the revised manuscript.

We rearranged the figures and the corresponding texts by moving original Figure 6, Microtubules influence the magnitude of diastolic crossbridges, to become revised Figure 5.

We also discussed that the mechanical contribution of microtubular stiffness may not have been fully captured under our experimental conditions, potentially because microtubules predominantly contribute to the viscous rather than elastic component of cellular stress (Loescher et al., 2023; Robison et al., 2016). Viscous stress is dependent on stretch velocity and is more pronounced at longer SLs (Chung et al., 2011a, Chung et al., 2011b). In this study, however, cells were stretched only to an SL of 2.15-2.2 μm to avoid compromising attachment to the glass rods. Please refer to lines 476-482 of the revised manuscript.

4.) The proposed link between elevated peak systolic calcium levels and diastolic XB contribution in HFpEF males also feels insufficiently justified, particularly given that the authors measure no change in diastolic calcium levels or myofilament calcium sensitivity. The same goes for proposed links between microtubule changes and changes to EC coupling/calcium levels, particularly without any measurements of ECC +/- colchicine.

Such speculation should be moved at least out of the results section, and more carefully considered in the discussion.

<u>Response.</u> We have removed statements from the Results section regarding the proposed link between elevated peak systolic Ca^{2+} levels and diastolic crossbridge contribution, as well as the relationship between microtubule changes and alterations in excitation-contraction coupling and Ca^{2+} handling.

Minor:

1. Please consider adding more contrast between groups for figure presentation (e.g. color the data points, bolder lines, etc) - sometimes it is difficult to discriminate the groups.

<u>Response.</u> We thank the reviewer for this helpful suggestion. We have revised the figures by adding more distinct colors for data points, and increasing the size of data points and line thickness where appropriate. We have also revised the original Figure 5C (revised Figures 6C) by separating the data into four individual panels representing the four experimental groups. Additionally, the original Figure 7 has been divided into revised Figures 7 and 8 to improve clarity and presentation.

2. Figure 2E seems to be missing x-axis labels to define groups.
Response. We thank the reviewer for noticing this oversight. We have revised Figure 2E to include the appropriate x-axis labels.

We thank the reviewer again for these insightful and constructive comments.

Reviewer #2 *(Comments to the Authors):*

This is an interesting work. Overall the findings are important for the field however, I do have a few concerns to address:

Response. We thank the reviewer for the positive assessment of our work and for recognizing the importance of our findings for the field. We appreciate the reviewer's careful evaluation of our manuscript and the opportunity to address the concerns raised. We have carefully considered each point and revised the manuscript accordingly. Detailed responses to each comment are provided below.

MAJOR

1.) Isn't it reductionist to restrict diastolic stiffness to three components. Authors should consider other contributors.

Response. We agree that, in addition to crossbridge activity, microtubules, and titin, multiple other factors contribute to cardiomyocyte diastolic stiffness. Accordingly, we have revised the Introduction to include the roles of desmin and the sarcolemma, and discussed the contribution of actin to sarcomere stiffness in the Discussion, as shown below.

"In addition to these primary contributors, intermediate filaments such as desmin, which anchor the microtubule network to the Z-disc region of the sarcomere, and the sarcolemma, also contribute to overall diastolic mechanical properties (Loescher et al., 2023)." Please refer to the revised manuscript lines 122-125.

"In addition to titin, recent work by Loescher et al. has shown that actin also contributes to cardiomyocyte viscoelastic properties, both through interactions with titin and via its intrinsic

viscoelasticity, independent of titin (Loescher et al., 2023). This finding aligns with proteomic analyses in HFpEF-like rat models, and human HFpEF myocardium, which revealed significant alterations in actin filament-binding, actin-organizing proteins, and actin cytoskeleton structures (Soetkamp et al., 2021, Jani et al., 2025). Together, these suggest that actin may play a more prominent role in regulating sarcomere passive properties than previously recognized and warrant further investigation." Please refer to lines 495-502 of the revised manuscript.

2.) Is it a real model of human HFpEF or diastolic dysfunction? Authors should be cautious

<u>Response.</u> We agree with the reviewer that the 2-hit model does not fully recapitulate all features of clinical HFpEF in humans. To address this limitation, we have revised the terminology throughout the manuscript and figure legends, replacing "HFpEF" with "HFpEF-like." However, for simplicity and figure readability, we retained the term "HFpEF" in the figure labels.

We also discussed the phenotypic variability and limitations of this model in the discussion section. Please refer to lines 506-534 of the revised manuscript.

3.) Age is not addressed at all (rationale, design, results...). This is important especially considering the model used.

<u>Response.</u> We apologize for this lack of clarity. We have described in the methods the age of the mice used as follows:

"Ovary-intact menopause was induced in a subgroup of female mice, by 4-vinylcyclohexene dioxide (VCD) intraperitoneal injection at 160 mg/kg daily for 20 days (Konhilas et al., 2020, Brooks et al., 2016). VCD injection began at 6 weeks of age." Please refer to lines 162-164 of the revised manuscript.

"HFpEF-like condition was induced using a 2-hit regimen (Schiattarella et al., 2019). The 2-hit protocol was initiated in both male and female mice at 3.5 months of age. For the 2 hit, mice were fed with a high-fat diet (D12492, Research Diet Inc) and L-NAME 0.5g/L (Nω-Nitro-L-arginine methyl ester; nitric oxide synthase inhibitor) in drinking water, continuing for 4 months." Please refer to lines 172-179 of the revised manuscript.

Thus, at the endpoint assessment, the mice were approximately 8-10 months of age, corresponding to middle-aged adulthood rather than aging. This, in part, explains why the model does not fully recapitulate the clinical HFpEF phenotype in humans. We also attempted to induce

the 2-hit HFpEF regimen in aged mice (~20 months old); however, this approach resulted in substantial variability in phenotype development, including failure to develop obesity in some animals as well as the occurrence of anemia or dermatitis in others. Therefore, although aging is an important contributor to HFpEF pathophysiology, aged mice were not included in the present study in order to maintain a more consistent and interpretable experimental model.

MINOR

Intro:

1st paragraph: 'etc' is not very scientific and should be refined properly.

Response. We have modified the sentences to remove the use of “etc.”, as follows.

“In the heart, the inflammatory cytokines cause deleterious effects on various organelles of the cardiomyocytes, including but not limited to the protein quality control system, mitochondria, Ca^{2+} handling proteins, cytoskeleton, and sarcomeres.” Please refer to lines 85-90 of the revised manuscript.

3rd paragraph: Overall, it is not easy to read. Also, please mention human data on microtubules.

Response. We have reorganized the original third paragraph by dividing it into three shorter paragraphs to improve readability.

In addition, we incorporated several new citations from published studies investigating microtubules in human myocardium into the Introduction, as follows.

“Microtubules are upregulated and undergo detyrosination, a PTM that increases their stiffness, in the myocardium of human end-stage heart and a rat model of HFpEF (Chen et al., 2018, Tsutsui et al., 1993, Eaton et al., 2024, Caporizzo et al., 2020). Restoration of tubulin tyrosination improves diastolic function in in vivo animal models and isolated human cardiomyocytes (Chen et al., 2020, Pietsch et al., 2024, Eaton et al., 2024).” Please refer to lines 111-116 of the revised manuscript.

4th paragraph: The rationale for studying sex differences is not presented in depth, and the hypothesis is not backed up by any references or justification.

Response. This work investigates the relative contributions of diastolic stress determinant, in both male and female mice, and identified significant sex-related differences. However, we did not examine in detail the molecular mechanisms underlying these observed sex differences. Thus, in

response to the reviewer's comment, we are considering modifying the title from "Sex Differences in Diastolic Contributors in Cardiomyocytes of the Cardiometabolic HFpEF Mouse Model" to "Diastolic Contributors in Cardiomyocytes of a Cardiometabolic HFpEF-Like Mouse Model." We have also removed the hypothesis statement from paragraph 4 of the original manuscript.

5th-6th-7th paragraph: The part on the 2-hit model is difficult to read with lots of repetition and should be put in the context of other models.

Response. We thank the reviewer for this helpful suggestion, which improves clarity and readability for the readers. We have removed repetitive statements and simplified the explanation describing why postmenopausal status was induced in female mice prior to application of the two-hit protocol. Please refer to lines 129-134 of the revised manuscript.

Methods:

A table summarizing cardiac function in the various groups is needed.

Response. In response to the reviewer's suggestion, we have added a supplementary table summarizing the echocardiographic and pressure-volume analysis parameters for all four experimental groups. Please refer to Supplementary Table 1 in the supplementary material.

Results:

Choice of SLs in general is unclear.

Response. In this study, intact cardiomyocytes were stretched only to an SL of 2.15-2.2 μm to avoid compromising cell attachment to the glass rods during mechanical measurements. Importantly, the target SL of 2.15 μm falls within the physiological diastolic SL range of the mouse left ventricle.

To clarify this point, we added the following sentence to the Results section: "This target SL lies within the physiological diastolic SL range of the mouse left ventricle (approximately 1.8-2.2 μm) (Chung and Granzier, 2011, Kobirumaki-Shimozawa et al., 2018))." Please refer to lines 263-265 of the revised manuscript.

Link between the individual findings are not clear as approaches are descriptive rather than mechanisitic.

Response. We thank the reviewer for this important comment. We agree that the present study is primarily descriptive and focused on identifying the relative contributions of different determinants

of diastolic dysfunction; however, it did not cover the mechanistic pathways linking the individual observations.

We acknowledge this limitation in the revised manuscript. Specifically, we added the following statement: “The present study focused on identifying the relative contributions of different determinants of diastolic dysfunction in this HFpEF-like model. While our findings provide physiological insights and establish associations among these contributors, the study did not cover the mechanistic pathways linking individual observations. Future studies are needed to investigate the molecular and cellular mechanisms underlying these findings, as well as their sex-specific differences”. Please refer to lines 550-554 of the revised manuscript.

Again, we thank the reviewer for these insightful and constructive comments.

Dr. Mei Methawasin
University of Missouri
Medical Pharmacology and Physiology
1 Hospital Dr
MA415 MSB
Columbia, MO 65212

Re: 202614028R1

Dear Dr. Methawasin,

I am pleased to let you know that your manuscript, titled "Diastolic contributors in cardiomyocytes of a cardiometabolic HFpEF-like mouse model." is scientifically acceptable for publication in Journal of General Physiology. Formal acceptance will follow when it is modified in accordance with the referees' remarks and our editorial policies.

Please note items that need attention are listed at the bottom of this email (under 'manuscript formatting checklist'). Your manuscript should be a double-spaced MS Word file and include editable tables, if appropriate.

Please submit your final files via this link:
Link Not Available

Thank you for choosing to publish your research in JGP and please feel free to contact me with any questions.

Sincerely,

Olaf S. Andersen, M.D.
On behalf of Journal of General Physiology

Journal of General Physiology's mission is to publish mechanistic and quantitative molecular and cellular physiology of the highest quality; to provide a best in class author experience; and to nurture future generations of independent researchers.

Manuscript formatting checklist:
- MS Word document of text needed (including editable tables)
- MS Word document of supplemental text needed, if applicable (including figure legends and editable tables)
- Brief Statement describing supplementary information needed, if applicable (in subsection at end of Materials & Methods)
- Please include a data availability statement preceding the Acknowledgments section. Please see https://rupress.org/jgp/pages/editorial-policies#data-availability-statement
- Figures created at sufficient resolution and in acceptable format (including supplemental if applicable). If working in Illustrator, we prefer .ai or .eps file format. If working in Photoshop please use 600dpi/1000dpi .tiff or .psd file format. Minimum resolution at estimated print size: Minimum resolution for all figures is 600 dpi. For figures that contain both photographs and line art or text, 600 dpi is highly recommended. Figures containing only black and white elements (line art, no color, and no gray) should be 1,000 dpi. Maximum figure size is 7 in wide x 9 in high (17.5 x 22.8 cm) at the correct resolution. https://jgp.rupress.org/fig-vid-guidelines
- Supplemental figures, if any, conforming to same guidelines as manuscript figures (noted above)
- If images resemble one from a prior publications, the author must seek permissions (to reproduce or adapt) from the original publisher. [You can resubmit your paper while waiting to hear back from the original publisher but please keep us updated]
- All authors must complete a disclosure form prior to acceptance. A link to complete the form has been sent to all coauthors. Please provide the editorial office with updated email addresses if necessary

---

Reviewer #1 (Comments to the Authors):
The authors have thoughtfully addressed reviewer comments, and the revised manuscript is improved. I have no further concerns.

Reviewer #2 (Comments to the Authors):
Thank you for revising the ms and taking the comments into consideration
