# Supplementary material for: Diastolic contributors in cardiomyocytes of a cardiometabolic HFpEF-like mouse model
Source: J Gen Physiol. 2026 Aug 25;158(6):e202614028. doi: 10.1085/jgp.202614028 (PMC13505484; doi:10.1085/jgp.202614028)
Supplement: Supplementary file 2 [file jgp_202614028_tables1.docx]

**Supplementary Table**

|  | Male  Ctrl | Male  HFpEF-like | Female  Ctrl | Female  HFpEF-like | 2-way ANOVA | | |
| --- | --- | --- | --- | --- | --- | --- | --- |
|  |  |  |  |  | inter-  action | HFpEF | sex |
|  |  |  |  |  |  |  |  |
| BW (gm) | 32.2±5.2 | 47.8±3.7**** | 24.4±3.1**** | 45.8±4.0****^,####^ | ** | **** | **** |
| **Echocardiography** | n=18 | n=23 | n=18 | n=30 |  |  |  |
| Heart Rate (bpm) | 443±56 | 421±47 | 456±58 | 463±44^$^ | ns | ns | * |
| EF (%) | 41.3±7.0 | 46.2±3.8 | 43.9±6.8 | 45.5±7.6 | ns | * | ns |
| stroke volume (μL) | 36.3±8.1 | 42.3±6.2 * | 33.3±6.6 | 33.2±6.9^$$$$^ | * | ns | *** |
| CO (mL/min) | 16.0±3.8 | 17.3±3.7 | 15.3±4.0 | 15.2±3.8 | ns | ns | ns |
| WT;d (mm) | 0.61±0.08 | 0.69±0.08 * | 0.57±0.05 | 0.68±0.08*^,####^ | ns | **** | ns |
| WT;s (mm) | 0.85±0.10 | 0.97±0.10 ** | 0.81±0.09 | 0.93±0.12^##^ | ns | **** | ns |
| LV EDV (μL) | 87.8±11.6 | 91.4±10.7 | 76.3±11.8** | 72.8±8.5****^, $$$$^ | ns | ns | **** |
| Eccentricity | 3.65±0.53 | 3.25±0.41* | 3.66±0.46 | 3.04±0.38****^,####^ | ns | **** | ns |
| LA dimension [mm] | 2.37±0.28 | 2.68±0.30 ** | 2.09±0.21** | 2.44±0.24^####,$$^ | ns | **** | **** |
| MV IVRT (msec) | 16.5±3.3 | 18.2±2.8 | 17.2±2.8 | 19.1±2.9* | ns | ** | ns |
| LV MPI | 0.61±0.12 | 0.66±0.12 | 0.60±0.09 | 0.64±0.08 | ns | ns | ns |
| E decel time (ms) | 24.1±3.8 | 19.9±3.3 ** | 22.3±5.5 | 18.8±3.0***^,#^ | ns | **** | ns |
| MV e' (mm/sec) | -17.32±2.84 | -15.51±3.14 | -18.6±4.9 | -15.9±3.4 | ns | ** | ns |
| MV E/A | 1.97±0.65 | 2.17±0.73 | 1.94±0.48 | 1.96±0.48 | ns | ns | ns |
| MV E/e´ | 29.3±6.3 | 38.5±8.2 *** | 29.7±7.3 | 35.8±6.5*^,#^ | ns | **** | ns |
| **Pressure-volume analysis** | n=13 | n=14 | n=14 | n=15 |  |  |  |
| ESP (mmHg) | 82.0±6.6 | 112.3±9.9**** | 78.1±7.5 | 104.9±12.6****^,####^ | ns | **** | ns |
| EDP (mmHg) | 3.86±1.93 | 7.90±2.89*** | 2.54±1.41 | 5.24±2.87^#,$^ | ns | **** | ** |
| dPmax (mmHg/s) | 6167±1500 | 7409±1217 | 6488±1241 | 7388±1178 | ns | ** | ns |
| dPmin (mmHg/s) | -5910±1044 | -7366±1381* | -5876±944 | -6849±1489 | ns | *** | ns |
| SV (µl) | 37.6±6.0 | 44.4±8.9 | 40.5±8.7 | 38.4±7.1 | * | ns | ns |
| CO (mL/min) | 15.1±2.6 | 18.6±4.9 | 17.9±4.7 | 15.3±3.0 | ** | ns | ns |
| Tau (msec) | 15.0±2.5 | 18.7±3.7* | 14.8±2.6 | 18.4±3.7*^,#^ | ns | **** | ns |
| Ea (mmHg/µl) | 2.26±0.39 | 2.68±0.69 | 2.01±0.38 | 2.93±0.83*^,###^ | ns | *** | ns |
| Ees (mmHg/µl) | 2.66±0.97 | 4.11±1.60* | 3.51±1.38 | 3.70±1.04 | ns | * | ns |
| PRSW (mmHg) | 66.6±17.4 | 83.7±11.4** | 72.0±8.7 | 77.5±11.0 | ns | ** | ns |
| EDPVR (mmHg/µl) | 0.020±0.008 | 0.039±0.02* | 0.026±0.01 | 0.049±0.021***^,##^ | ns | **** | ns |
|  |  |  |  |  |  |  |  |

**Supplementary Table1.** **Echocardiographic parameters.** The abbreviations used include BW (body weight), EF (ejection fraction), CO (Cardiac Output), WT;d (wall thickness in diastole), WT;s (wall thickness in systole), LVEDV (LV end-diastolic volume), Eccentricity (LVID;d / (LVPW;d + LVAW;d)), LA (left atrium), IVRT (Isovolumic Relaxation Time), MPI (myocardial performance index), E decel time (Mitral E deceleration time), MV (mitral valve), E (early diastolic filling), A (late diastolic filling), and e’ (early diastolic velocity of mitral annulus movement). **Pressure-volume analysis parameters.** The abbreviations used include ESP (end-systolic pressure), EDP (end-diastolic pressure), dPmax (the maximum rate of pressure rise), dPmin (the maximum rate of pressure decrease), SV (stroke volume), CO (cardiac output), Tau (LV relaxation–time constant), Ea (effective arterial elastance), Ees (end-systolic elastance, equivalent to ESPVR), PRSW (preload recruitable stroke work), EDPVR (end-diastolic pressure-volume relation).

The data is presented as means ± SD. Statistical significance is indicated as * for p≤0.05, ** for p≤0.01, *** for p≤0.001, **** for p≤0.0001. The analysis was conducted using two-way ANOVA with post hoc testing using Tukey’s method. * indicates a significant difference relative to male-Ctrl, # indicates a significant difference relative to female-Ctrl, $ indicates a significant difference relative to male-HFpEF.The significance between female- Ctrl vs male-HFpEF is not shown for simplicity.
